# The shortcut of mycobacterial interspersed repetitive unit-variable number tandem repeat typing for *Mycobacterium tuberculosis* differentiation

**DOI:** 10.3389/fmicb.2022.978355

**Published:** 2022-09-08

**Authors:** Shima Hadifar, Mansour Kargarpour Kamakoli, Sana Eybpoosh, Mehran Nakhaeizadeh, Mohammad Ali Kargarpour Kamakoli, Nasim Ebrahimifard, Abolfazl Fateh, Seyed Davar Siadat, Farzam Vaziri

**Affiliations:** ^1^Department of Mycobacteriology and Pulmonary Research, Pasteur Institute of Iran, Tehran, Iran; ^2^Microbiology Research Center, Pasteur Institute of Iran, Tehran, Iran; ^3^Department of Epidemiology and Biostatistics, Research Centre for Emerging and Reemerging Infectious Diseases, Pasteur Institute of Iran, Tehran, Iran; ^4^Modeling in Health Research Center, Institute for Futures Studies in Health, Kerman University of Medical Sciences, Kerman, Iran; ^5^Department of Biostatistics and Epidemiology, School of Public Health, Kerman University of Medical Sciences, Kerman, Iran; ^6^Department of Mechanical Engineering, Quchan University of Technology, Quchan, Iran

**Keywords:** *Mycobacterium tuberculosis*, MIRU-VNTR, LASSO regression, accumulation of the percentage differences, genotyping

## Abstract

The 24-loci mycobacterial interspersed repetitive unit-variable number tandem repeat (MIRU-VNTR) genotyping has been used as an international standard method for *Mycobacterium tuberculosis (Mtb)* genotyping. However, different optimized VNTR loci sets for improving the discrimination of specific *Mtb* genotypes have been proposed. In this regard, we investigated the efficacy of accumulation of the percentage differences (APDs) compared with the least absolute shrinkage and selection operator (LASSO) regression strategy to identify a customized genotype-specific VNTR loci set which provides a resolution comparable to 24-loci MIRU-VNTR in divergent *Mtb* populations. We utilized Spoligotyping and 24-loci MIRU-VNTR typing for genotyping 306 *Mtb* isolates. The APD and LASSO regression approaches were used to identify a customized VNTR set in our studied isolates. Besides, the Hunter-Gaston discriminatory index (HGDI), sensitivity, and specificity of each selected loci set were calculated based on both strategies. The selected loci based on LASSO regression compared with APD-based loci showed a better discriminatory power for identifying all studied genotypes except for T genotype, which APD-based loci showed promising discriminative power. Our findings suggested the LASSO regression rather than the APD approach is more effective in the determination of possible discriminative VNTR loci set to precise discrimination of our studied *Mtb* population and may be beneficial to be used in finding reduced number loci sets in other *Mtb* genotypes or sublineages. Moreover, we proposed customized genotype-specific MIRU-VNTR loci sets based on the LASSO regression and APD approaches for precise *Mtb* strains identification. As the proposed VNTR sets offered a comparable discriminatory power to the standard 24 MIRU-VNTR loci set could be promising alternatives to the standard genotyping for using in resource-limited settings.

## Introduction

Understanding and monitoring the patterns and dynamics in the population structure of *Mycobacterium tuberculosis* (*Mtb*), as one of the deadly infectious agents, is imperative for epidemiological and biological purposes. Molecular typing methods play an important role in achieving this purpose and provide powerful tools to measure local and global tuberculosis (TB) surveillance (Allix-Béguec et al., 2008; Hachisu et al., 2013). Over the previous decade, different polymerase chain reaction (PCR)-based genotyping methods, such as the 24-loci mycobacterial interspersed repetitive unit-variable number tandem repeat (MIRU-VNTR) typing and Spoligotyping have been widely used as valuable tools for identification of *Mtb* strain (Allix-Béguec et al., 2008; Ei et al., 2016). Although Whole-Genome Sequencing (WGS) has been suggested as a proxy to trace *Mtb* transmission and provides a comprehensive identification; however, due to several limitations have not been in resource-limited settings compared with PCR-based methods (Satta et al., 2018).

MIRU-VNTR typing is considered a promising genotyping method, providing a comparable or superior resolution compared with other PCR-based genotyping methods like IS6110-RFLP and Spoligotyping depending on the selected VNTR loci set (De Beer et al., 2012; de Beer et al., 2013). Generally, MIRU-VNTR loci are selected based on the discrimination power of each locus, which can result in genotyping errors for different *Mtb* families due to the different genetic backgrounds of the populations and reduce discrimination power based on specific genotypes (Murase et al., 2008; Luo et al., 2014). It has been proposed that an optimized locus set based on the accumulation of the percentage differences (APDs) approach can efficiently discriminate specific genotypes (Beijing, NEW-1, and CAS) (Pan et al., 2017; Hadifar et al., 2019a). Accordingly, in this study, we adopted another approach to investigate the capacity and efficacy of APD compared with the least absolute shrinkage and selection operator (LASSO) regression approach to identify a customized genotype-specific MIRU-VNTR loci set, which provides a resolution comparable to 24-loci VNTR in divergent *Mtb* population.

## Materials and methods

### Clinical isolates collection

A total of 306 *Mtb* isolates were randomly collected from who had been diagnosed with pulmonary TB from January 2017 to January 2019. All patients had no history of anti-TB treatment. None of the samples were collected from the same patient. In addition, all *Mtb* isolates were confirmed by PCR for IS6110. Culture-negative pulmonary TB cases were also excluded. This study was approved by the Ethics Committee of the Pasteur Institute of Iran. All experiments were performed in accordance with guidelines approved by Pasteur Institute International networks. Ethical reviews and informed consent approval were granted by the Ethical Committee of the Pasteur Institute of Iran. Informed consent was obtained from all patients in this study. The results of this research did not influence TB patients’ treatment.

### Twenty-four-loci mycobacterial interspersed repetitive unit-variable number tandem repeat typing

The extraction of *Mtb* genomic DNA was performed by a PROBA-NK DNA extraction kit (DNA Technology Company, Moscow, Russia) according to the manufacturer’s instructions and was used for the 24-loci MIRU-VNTR genotyping method as described previously (Supply et al., 2006).

### Spoligotyping

Spoligotyping commercial kit (Mapmygenome Genomics Company, India) was used for analyzing all *Mtb* isolates based on the Spoligotyping protocol described by Kamerbeek et al. (1997).

### Statistical analysis

*Mtb* genotypes were initially identified with the MIRU-VNTR technique and confirmed with the Spoligotyping method. The MIRU-VNTR and Spoligotyping data were analyzed by the MIRU-VNTRplus web server^1^ and SITVIT-WEB online tool^2^ (Demay et al., 2012), respectively. In addition, TBminer databases (Azé et al., 2015) were used to analyze and confirm the results.

Simpson’s diversity index for each VNTR locus and VNTR loci in combination, as described by Hunter and Gaston (1988), was calculated using the online tool at http://www.comparingpartitions.info/index.php. Based on the Hunter-Gaston discriminatory index (HGDI) score, each locus showed a high (*h* > 0.6), moderate (0.3 ≤ *h* ≤ 0.6), and poor (*h* < 0.3) discriminative power (Sola et al., 2003).

The data set consisted of variables with more than 30 percent missing values. We checked the mechanisms of missing values which were completely at random. Therefore, the missing values were imputed using Multiple Imputations *via* the Chained Equations (MICE) algorithm. LASSO regression method was used to provide a reduced, and hence, more cost-effective set of loci that could predict the *Mtb* genotypes with reasonable accuracy. The LASSO regression model, which is acknowledged to be suitable for high dimensional data regression analysis (Sauerbrei et al., 2007), is a regularization-based regression model that uses shrinkage. The lasso shrinks the coefficient estimates toward zero. It performs as a variable selection method and adds a penalty term (L1) to the cost function. Indeed, in lasso regression, we aim to find estimates of the coefficients β to βp that minimize RSS + $\lambda^{*}\,\sum_{j=1}^{p}\left|\beta_{j}\right|$ where RSS is the residual sum of squares. So, if λ = 0, then we have regular ordinary least squares regression. Accordingly, LASSO regression was performed to select the optimal set of VNTR loci for predicting *Mtb* genotypes.

Afterward, the predicted values, sensitivity, and specificity indices were computed and used for comparison with APD method. Sensitivity and specificity were used to evaluate the accuracy of a test that predicts dichotomous outcomes (e.g., logistic regression). Sensitivity evaluates model performance and measures a model’s capability to identify positive examples correctly. When sensitivity is used to evaluate model performance, it is often compared to specificity. Specificity measures the proportion of true negatives that the model correctly identifies. We assessed internal and external validity to validate the performance of LASSO regression. The fivefold cross-validation (CV) was used for evaluating internal validity. This approach randomly divides the data 5 times as training and test samples and runs the analysis on each fold. Then the sensitivity and specificity indices were calculated. External validity was tested by defining our dataset as a train set and an additional source of data as a test set, including the collection of 225 strains with their 24 MIRU-VNTR loci and genotype assignments provided from the MIRU-VNTRplus reference database (see text footnote 1) and a part of data from two previous studies (Homolka et al., 2008; Shuaib et al., 2022; Supplementary Table 2). Then the sensitivity and specificity indices were also calculated. Analyses were performed in R software.

To APD calculation in each VNTR locus, firstly, we categorized our MIRU-VNTR data into eight data groups, including Beijing and Non-Beijing, CAS and Non-CA, NEW-1 and Non-NEW-1, Haarlem and Non-Haarlem, Cameroon and Non- Cameroon, LAM and Non-LAM, T and Non-T, and Ural1 and Non-Ural1. Then, the frequency of repeat numbers (patterns) at any VNTR locus was determined in both groups of each category. Finally, the APD value for each VNTR locus in every category was calculated as follows:


$$A\,P\,D=1\,/\,2\,\sum_{J=1}^{S}\left|A\,j-B\,j\right|$$

Where s is the total number of distinct repeat patterns, Aj (Bj) is the percentage of A (B) category strains with the jth repeat pattern from all the strains of the A (B) category.

## Results

According to results of the standard 24-loci MIRU-VNTR genotyping with TBminer database, we identified eight genotypes as follows: 101 CAS1-Delhi (33.00%), 88 NEW-1 (28.76%), 56 Beijing (18.30%), followed by 16 isolates as Haarlem (5.22%), 16 isolates as Cameroon (5.22%), 12 isolates as T (3.92%), 9 isolates as Ural1 (2.94%), and 8 isolates as LAM genotype (2.61%). The Spoligotyping method showed a similar result to MIRU-VNTR data (Supplementary Table 1). HGDI of each VNTR locus was calculated for each genotype. According to the global HGDI, 8 VNTR loci showed a high discriminatory power including QUB26 (4052), MIRU10 (960), MIRU26 (2996), ETRA (2165), MTUB04 (424), MTUB21 (1955), MIRU16 (1644), and QUB11B (2163b); while VNTR MIRU24 (2687), MTUB29 (2347), MIRU02 (154), MIRU27 (3007), MIRU04 (580), and MIRU20 (2059) were poorly discriminative (HGDI < 0.3).

We previously defined a combination of loci for specific genotypes (NEW-1 and CAS1-Delhi) based on APD (Hadifar et al., 2019a). Here, we examined an additional strategy to select the customized genotype-specific MIRU-VNTR loci set based on LASSO regression and APD to improve the power of differentiation in the *Mtb* population. Besides, HGDI, sensitivity, and specificity of each selected loci set were investigated based on the strategies. As shown in Table 1, selected loci based on LASSO regression approach showed a better discriminatory power for identifying all the studied genotypes (especially in locally dominant *Mtb* genotypes) compared with those based on the APD approach except for T genotype, which APD-based loci showed the better result. Besides, external and internal validity results for the LASSO regression approach showed proper and comparable sensitivity and specificity in most of the studied genotypes (Table 1).

**TABLE 1:** HGDI values of proposed genotype-specific MIRU-VNTR loci based on APD and LASSO regression approaches.

Spoligotyp efamily	Loci(APD)	HGDI	Sensitivity%	Specificity%	Loci(LASSO)	HGDI	Sensitivity% ^a^	Specificity% ^a^	Sensitivit% ^b^	Specificity% ^b^	N
Global	24loci	0.964	−	−	24loci	**0.964**			−	−	306
CAS	ETRC (577),MIRU10(960),MIRU 16 (1644),MIRU26 (2996),MTUB21(1955)	0.923	**83.1**	**99.5**	ETRC(577),MIRU10(960),MIRU16(1644),MIRU26(2996),MIRU39(4348)	**0.956**	98	93.7	100	94.4	101
NEW−1	ETRB(2461),MIRU10(960),MTUB34(3171),MTUB04(424),ETRA(2165)	0.92	**79.3**	**100**	MIRU10(960),MTUB21(1955),ETRB(2461),MTUB34(3171),MIRU31(3192)	**0.943**	98.9	96.8	100	94.1	88
Beijing	QUB11B(2163b),MIRU10(960),MTUB30(2401),ETRA(2165),MIRU31(3192)	0.876	**80.5**	**98.9**	MTUB21(1955),QUB11B(2163b),ETRA(2165),MTUB30(2401),MIRU31(3192)	**0.934**	96.4	98.4	100	83.9	56
Haarlem	MIRU10(960),MTUB21(1955),MTUB30(2401),MIRU26(2996)	0.933	**25**	**100**	MIRU16(1644),MTUB21(1955),MTUB30(2401),MIRU26(2996)	**0.952**	81.2	67.6	96.4	85.8	16
Cameroon	MIRU10(960),MTUB21(1955),QUB11B(2163b),MIRU26(2996),MIRU31(3192),QUB26(4052)	0.859	**77.8**	**97.7**	MIRU02(154),MIRU16(1644),MTUB21(1955),QUB11B(2163b),MTUB30(2401),ETRB(2461),MIRU24(2687),QUB26(4052)	**0.934**	93.8	78.3	100	61.6	16
T	ETRC(577),MIRU40(802),MIRU10(960),MTUB21(1955),MIRU23(2531),MIRU26(2996)	0.949	**100**	**98.3**	MTUB04(424),MIRU40(802),MIRU10(960),MTUB21(1955),MIRU23(2531),MTUB39(3690),QUB26(4052)	**0.917**	100	71.1	62.9	52.1	12
Ural1	MIRU40(802),MIRU10(960),MIRU16(1644),MTUB21(1955),MTUB30(2401),MIRU26(2996),QUB4156(4156)	0.924	**80**	**100**	MIRU02(154),MTUB04(424),MIRU10(960),MTUB30(2401),MIRU26(2996),QUB4156(4156)	**0.94**	100	97.6	100	90	9
LAM	MIRU10(960),MTUB21(1955),QUB11B(2163b),,ETRA(2165),MIRU23(2531)	0.912	**83.3**	**98.7**	MIRU20(2059),ETRA(2165),MIRU23(2531),MIRU31(3192)	**0.93**	100	92.3	90	91.7	8

Internal validation: The 5-fold cross-validation (CV) was used for evaluating internal validity. This approach randomly divides the data 5 times as training and test samples and runs the analysis on each fold. External validation: External validity was tested by defining our dataset as a train set and an additional source of data as a test set.

In the CAS-Delhi population, a combination of five VNTR loci [ETRC (577), MIRU10 (960), MIRU16 (1644), MIRU26 (2996), and MTUB21 (1955)] was found as a proper set based on the descending order of the APD values (Table 2). According to the LASSO regression value, five VNTR loci [ETRC (577), MIRU10 (960), MIRU16 (1644), MIRU26 (2996), MIRU39 (4348)] were identified as the customized genotype-specific MIRU-VNTR loci. Four out of five LASSO regression-based loci were similar to APD-based loci [VNTR ETRC (577), MIRU10 (960), MIRU16 (1644), and MIRU26 (2996)].

**TABLE 2:** The APDs of 24 loci VNTR in all the studied genotypes.

MIRU-VNTR locus	Beijing and non-Beijing	CAS and non-CAS	NEW-1 and non-NEW-1	Haarlem and non-Haarlem	Cameroon and non-Cameroon	LAM and non-LAM	T and non-T	Ural1 and non-Ural1
	**APD%**	**APD%**	**APD%**	**APD%**	**APD%**	**APD%**	**APD%**	**APD%**
**MIRU02** **(154)**	3.01	1.95	5.74	4.53	18.41	11.49	5.44	32.32
**MTUB04** **(424)**	47.27	45.61	63.38	29.66	43.62	23.95	38.69	41.92
**ETRC** **(577)**	41.86	93.61	54.36	24.33	43.62	24.92	74.40	40.24
**MIRU04** **(580)**	7.21	2.33	2.44	8.73	8.10	5.29	8.16	3.87
**MIRU40** **(802)**	23.58	14.46	15.44	27.54	29.66	29.19	55.53	72.73
**MIRU10** **(960)**	76.24	79.65	92.92	48.02	67.54	59.56	68.79	78.11
**MIRU16** **(1644)**	32.49	74.39	44.70	32.61	29.31	32.55	22.87	57.24
**MTUB21** **(1955)**	36.00	54.95	46.80	58.00	77.16	51.51	60.88	87.21
**MIRU20** **(2059)**	7.11	1.30	1.90	11.92	6.94	24.29	13.35	31.14
**QUB11B** **(2163b)**	89.04	41.38	35.92	34.68	46.06	58.18	26.70	29.29
**ETRA** **(2165)**	59.35	24.27	59.25	29.01	30.86	74.96	39.63	25.42
**MTUB29** **(2347)**	1.21	3.64	2.38	4.01	3.62	3.52	6.12	20.37
**MTUB30** **(2401)**	68.50	8.40	46.56	45.86	35.00	33.72	34.52	71.89
**ETRB** **(2461)**	33.23	46.03	94.06	25.82	30.65	36.24	20.92	30.64
**MIRU23** **(2531)**	13.80	12.08	20.66	5.09	23.71	88.59	70.28	17.85
**MIRU24** **(2687)**	0.89	1.22	1.15	0.86	12.33	0.84	0.85	0.84
**MIRU26** **(2996)**	28.89	57.56	38.50	40.32	53.41	38.00	59.18	94.28
**MIRU27** **(3007)**	16.06	3.52	6.69	7.84	9.83	11.66	9.69	17.68
**MTUB34** **(3171)**	36.40	33.56	69.23	24.78	21.49	21.14	21.77	13.47
**MIRU31** **(3192)**	57.09	37.58	54.40	29.89	46.38	35.40	22.87	43.94
**MTUB39** **(3690)**	5.43	6.96	14.36	7.59	26.38	25.67	46.51	26.09
**QUB26** **(4052)**	41.06	33.13	54.75	12.31	59.03	37.92	42.18	49.16
**QUB4156** **(4156)**	11.34	25.07	28.18	11.06	14.89	16.07	22.28	53.87
**MIRU39** **(4348)**	34.55	48.89	44.92	37.20	37.20	42.62	34.52	31.31

The VNTR loci set obtained based on LASSO regression showed the similar level of discrimination as the 24 loci reference method (HGDI: 0.956). Besides, the estimated sensitivity and specificity of the selected loci set based on LASSO regression were more significant compared with the APD results (Table 1).

In the NEW-1 population, the VNTR loci set of ETRB (2461), MIRU10 (960), MTUB34 (3171), MTUB04 (424), and ETRA (2165) were identified based on the APD approach. The APD value of the selected loci was more than 59% (Table 2). Based on the LASSO regression approach, the VNTR loci set of MIRU10 (960), MTUB21 (1955), ETRB (2461), MTUB34 (3171), and MIRU31 (3192) were identified as a customized loci set. According to the HGDI value of each set, the loci set obtained based on LASSO regression exhibited the promising discriminative power in this genotype (HGDI: 0.943). The specificity and sensitivity values of the LASSO-based loci also were more significant compared with the APD results (Table 1).

In the Beijing population, as shown in Tables 1, 2, the effective VNTR loci for the discrimination of the genotype had an APD value of more than 50% consistent with our previous result (Hadifar et al., 2019a). Based on LASSO regression, MTUB21 (1955), QUB11B (2163b), ETRA (2165), MTUB30 (2401), and MIRU31 (3192) loci were identified as a customized loci set, for identification of Beijing genotype with a promising discriminatory value (HGDI: 0.934).

As depicted in Table 1, in other genotypes, including Haarlem, Cameroon, LAM, and Ural1, the LASSO-based loci set showed a higher HGDI value than the APD-based loci set. The values of sensitivity and specificity exhibited a variation between the two strategies in each genotype. In T genotype, HGDI value obtained based on APD approach showed promising discriminative power.

## Discussion

The 24-loci MIRU–VNTR genotyping has been used as an international standard method for *Mtb* genotyping. However, regarding a specific strain background and polymorphism which can affect the resolution of discrimination, various studies in the different geographical regions have tried to explore optimized VNTR sets for improving the standard typing method and offered different VNTRs combinations (Ali et al., 2007; Asante-Poku et al., 2014). In addition, previous studies have shown that a combination of VNTR loci selected based on the discrimination power method (HGDI) displayed a low capacity for a precise classification in endemic genotypes (Pan et al., 2017; Hadifar et al., 2019a). In this regard, we used two different approaches, including APD and LASSO regression to identify an optimized VNTR set in our studied *Mtb* population. We first established a specific genotype panel based on two utilized methods. In the next step, a comparison of the HGDI of each VNTR set with HGDI, sensitivity, and specificity of the reference method showed that LASSO regression compared with APD was more effective in discrimination of the studied *Mtb* genotype, especially for CAS, NEW-1, and Beijing genotypes.

Different studies showed that there is a remarkable genotypic diversity among *Mtb* population in Iran (Merza et al., 2010; Mansoori et al., 2018; Hadifar et al., 2019b,^2021^; Kochkaksaraei et al., 2019; Vaziri et al., 2019). According to the reports, certain genotypes, such as CAS, NEW-1, and Beijing are more medically important than other genotypes in this geographical region. These genotypes showed a high capacity for drug resistance acquisition (Sinkov et al., 2016). A more recent meta-analysis study using MIRU-VNTR typing and Spoligotyping data demonstrated that NEW-1 and CAS are the most dominant *Mtb* genotypes in Iran. Besides, Beijing was shown as the prominent genotype circulating among the multidrug-resistant *Mtb* population (Hadifar et al., 2021). Based on this diversity, an in-depth study for the precise identification of *Mtb* genotypes can be helpful for better management and control of their infection.

Some MIRU-VNTR-based studies have suggested a set of loci including MIRU10 (960), MIRU16 (1644), MIRU26 (2996), MIRU27 (3007), MIRU31 (3192), MIRU39 (4348), MIRU40 (802), MTUB21 (1955), and QUB26 (4052) are more discriminative in the standard 24 MIRU-VNTR loci panel for identification of different *Mtb* isolates circulating in Iran (Zamani et al., 2016; Mansoori et al., 2018). However, the described loci were also offered based on HGDI, which previously showed that selecting VNTR loci based on this index displayed a low capacity for a precise classification in endemic genotypes (Pan et al., 2017; Hadifar et al., 2019a).

We have previously reported that using the APD approach may facilitate efficient discrimination and clustering of NEW-1 and CAS populations (Hadifar et al., 2019a). In the present study, 5 optimized VNTR loci namely, ETRC (577), MIRU10 (960), MIRU16 (1644), MIRU26 (2996), and MIRU39 (4348) (with a sensitivity of 98% and a specificity of 93.7% in internal validity and sensitivity of 100%, and a specificity of 94,.4% in external validity) and five optimized loci, including ETRC (577), MIRU10 (960), MIRU16 (1644), MIRU26 (2996), and MTUB21 (1955) (with a sensitivity of 83.1% and a specificity of 99.5%) were identified based on LASSO regression and APD approaches for CAS genotype, respectively. The APD result was consistent with our previous study (Hadifar et al., 2019a), in which VNTR loci with APDs of more than 50% could efficiently discriminate the CAS and non-CAS genotypes. In addition, the selected loci were similar to those reported in our previous study (Hadifar et al., 2019a). Four VNTR loci, including ETRC (577), MIRU10 (960), MIRU16 (1644), and MIRU26 (2996) were also found in LASSO analysis. In a study by Rasoahanitralisoa et al. (2017) a combination of ten VNTR loci [MIRU10 (960), QUB4156 (4156), MTUB04 (424), MIRU16 (1644), MIRU40 (802), ETRC (577), MTUB39 (3690), QUB26 (4052), MIRU31 (3192), and QUB11B (2163b)] have been proposed as discriminative loci for CAS genotype combined principal component analysis (PCA) and HGDI methods, which only five loci (QUB4156 (4156), MIRU10 (960), MIRU16 (1644), MIRU40 (802), and MTUB04 (424) of the panel have been selected based on the PCA method. Overall, these findings suggest that the combination of VNTR ETRC (577), MIRU10 (960), and MIRU16 (1644), might be considered a preliminary marker that facilitates the identification of the CAS population with higher discriminatory power. In addition, we found the LASSO regression-based VNTR selection process offers a more helpful strategy for precise discrimination of CAS from the non-CAS population in different settings.

In the NEW-1 population, as a member of the Euro-American super lineage, five combinations of VNTR loci based on APD and five combinations of VNTR loci based on LASSO were found with a high capacity for efficient discrimination. In addition, a combination of VNTR MIRU10 (960), ETRB (2461), and MTUB34 (3171) which were similar between the two methods could be used a primary set for identifying the NEW-1 family. The efficient implementation of these methods for genotype data is helpful for the effective selection of VNTR loci for NEW-1 discrimination. However, based on the specificity of each selected set, we can suggest that using LASSO may be more effective than APD for discrimination of the NEW-1 population.

In Beijing population, as the third dominant genotype, a set of loci, which can more effectively classify this genotype were obtained based on the LASSO method, including VNTR MTUB21 (1955), QUB11B (2163b), ETRA (2165), MTUB30 (2401), and MIRU31 (3192). (With a sensitivity of 96.4% and a specificity of 98.4% in internal validity and sensitivity of 100%, and a specificity of 83.9% in external validity, HGDI: 0.934). Five loci set selected based on APD method also showed the high discriminatory power in this genotype (with a sensitivity of 80.5% and a specificity of 98.9%, HGDI: 0.876). In a previous study, 12 optimized loci were suggested for discrimination of Beijing genotype based on the combined PCA and HGDI methods. Among these loci, only five loci [QUB11B (2163b), MIRU26 (2996), QUB26 (4052), MTUB30 (2401), and ETRC (577)] were selected based on the PCA result (Rasoahanitralisoa et al., 2017). A different combination of loci has been described in Pan et al. (2017) study based on the APD method. It can be speculated that these discordances reflect the difference in the specific circulating sub-lineage, geographical region, VNTR loci to identify a suitable set, and sample size.

Other genotypes identified in our study were a part of the Euro-American super lineage, which is the most widely dispersed lineage. Different customized VNTR loci sets were offered based on both applied approaches to high effective discrimination of these groups. However, the selected loci sets based on both methods were reliable and promising in most of these genotypes, based on HGDI value, LASSO-based VNTR loci were more discriminative than the APD-based loci. Besides, no relevant study was found on the discriminative power of the loci combination based on APD, LASSO, and/or other statistical methods for Haarlem, Cameroon, LAM, and Ural1. Further investigations are crucial to accredit the efficiency of our implemented approaches in selecting discriminative loci in the other main *Mtb* genotypes and some genotypes in our study with small sample size like T. Another limitation of this study is that selection of VNTR loci was based on the predefined panel for standard 24 loci MIRU-VNTR typing, so evaluating additional loci could be beneficial to improve the efficiency of the selected customized loci set in discrimination of *Mtb* isolates in different settings. In addition, more *Mtb* genotypes should have been included in the current study.

## Conclusion

In summary, we proposed customized genotype-specific MIRU-VNTR loci sets based on the LASSO regression and APD approaches for precise *Mtb* strains identification. As the proposed VNTR set offered a comparable discriminatory power to the standard 24 MIRU-VNTR loci set could be promising alternatives to the 24-loci MIRU–VNTR genotyping for using in resource-limited-settings. Moreover, our findings suggested the LASSO regression rather than the APD approach is more effective in the determination of possible discriminative VNTR loci set to precise discrimination of our studied *Mtb* population and may be beneficial to be used in finding reduced number loci set in other *Mtb* genotypes or sublineages.

## Data availability statement

The original contributions presented in this study are included in the article/Supplementary material, further inquiries can be directed to the corresponding author/s.

## Ethics statement

The studies involving human participants were reviewed and approved by Pasteur Institute of Iran. The patients/participants provided their written informed consent to participate in this study.

## Author contributions

SH and FV wrote the manuscript. FV supervised the project. FV, SDS, and AF designed the project. SH, NE, and MKK performed laboratory work. SE, MN, and MAKK performed the statistical analysis. All authors have read and approved the final manuscript.

## Footnotes

1
http://www.miru-vntrplus.org

2
http://www.pasteur-guadeloupe.fr:8081/SITVIT_ONLINE/index.jsp
