# Supplementary material for: The shortcut of mycobacterial interspersed repetitive unit-variable number tandem repeat typing for Mycobacterium tuberculosis differentiation
Source: Front Microbiol. 2022 Sep 8;13:978355. doi: 10.3389/fmicb.2022.978355 (PMC9493315; doi:10.3389/fmicb.2022.978355)
Supplement: Supplementary file 2 [file Data_Sheet_2.pdf]

Supplementary Table S2: MIRU-VNTR of 225 *Mtb* strains used as a test set for external validity test

ID	Country of Isolation	Genotype	154	424	577	580	802	960	1644	1955	2059	2163b	2165	2347	2401	2461	2531	2687	2996	3007	3171	3192	3690	4052	4156	4348
05BR0205	estern Euro	CAS	2	4	2	2	3	6	3	6	2	2	4	4	2	2	5	1	4	3	3	4	3	8	3	3
10264/03	Germany	TUR	2	4	4	1	2	5	1	2	2	2	3	4	4	2	5	1	1	3	3	3	3	6	3	2
10438/01	Ghana	Cameroon	2	2	4	2	3	3	2	3	1	3	4	4	2	2	5	1	5	3	3	3	a	4	2	2
10439/01	Ghana	Cameroon	2	2	4	2	3	3	3	3	1	6	4	4	2	2	5	1	5	3	3	3	d	5	2	2
10445/01	Ghana	Cameroon	2	2	4	2	3	3	3	3	1	6	4	4	2	2	5	1	5	3	3	3	8	4	0	2
10446/01	Ghana	Cameroon	1	2	4	2	3	3	2	3	1	4	3	4	2	2	5	1	4	3	3	3	9	5	2	2
10459/03	Germany	NEW-1	2	1	4	2	3	2	3	3	2	3	3	4	2	1	5	1	5	3	3	3	3	7	2	2
10469/01	Ghana	T	2	2	3	2	5	3	3	4	2	3	3	4	4	2	5	1	4	3	2	3	3	6	1	3
10470/01	Ghana	T	2	2	3	2	6	3	3	4	2	3	3	4	4	2	5	1	4	3	2	3	3	6	1	3
10481/01	Ghana	Cameroon	2	2	4	2	3	3	3	3	1	4	2	4	2	2	5	1	5	3	3	3	9	4	2	2
10486/01	Ghana	T	2	2	3	2	6	3	3	4	2	3	3	4	4	2	5	1	4	3	2	3	3	6	1	3
10493/01	Ghana	T	2	2	3	2	6	3	3	3	2	4	3	4	4	2	5	1	4	3	2	4	3	6	1	3
10515/01	Ghana	T	2	2	3	2	6	3	3	4	2	3	3	4	4	2	5	1	4	3	2	3	3	6	1	3
10529/03	Germany	TUR	2	4	4	1	2	5	1	2	2	2	3	4	4	2	5	1	1	3	3	3	3	6	3	2
10581/03	Germany	LAM	1	1	2	2	4	4	3	3	2	2	2	4	1	2	5	1	5	3	3	2	2	5	2	2
11046/04	Germany	S	3	3	4	3	4	3	3	1	2	4	3	2	2	2	5	1	5	3	3	3	3	9	2	2
11051/03	Germany	EAI	2	2	4	4	3	4	2	5	2	2	7	3	2	5	5	2	2	3	3	5	4	5	1	3
11313/03	Germany	TUR	2	4	4	1	2	5	1	2	2	2	3	4	4	2	5	1	1	3	3	3	3	6	3	2
11359/03	Germany	EAI	2	2	4	5	4	5	3	3	1	b	8	3	2	5	6	2	2	3	3	5	6	6	1	3
12	Sierra Leone	T	2	2	2	2	4	3	1	2	2	3	3	4	2	2	5	1	5	3	3	3	5	5	2	2
12591/02	Germany	NEW-1	2	2	4	2	3	2	3	5	2	2	3	4	2	1	5	1	6	3	2	3	4	7	2	2
12637/02	Germany	Haarlem	2	2	3	2	3	5	3	3	2	4	2	2	4	2	3	1	5	3	3	3	3	7	3	2
12778/03	Germany	EAI	2	2	4	6	3	4	2	5	2	2	7	3	2	4	5	2	2	3	3	5	5	6	1	3
1417/02	Ghana	Cameroon	2	2	4	2	3	3	3	3	1	5	4	4	2	2	5	1	5	3	3	3	6	5	2	2
1428/02	Ghana	Cameroon	2	2	4	2	3	3	3	3	1	4	4	4	2	2	5	1	5	3	3	3	4	5	2	2
1438/02	Ghana	T	2	2	3	2	5	3	3	4	2	3	3	4	4	2	5	1	4	3	2	3	3	6	1	3
15	Sierra Leone	T	2	4	3	2	4	5	3	3	2	4	2	4	4	2	5	1	4	3	3	3	3	9	1	2
1521/99	Uganda	T	2	2	5	2	3	3	3	3	1	2	4	4	2	1	5	1	4	4	3	4	3	6	2	2
1571/99	Uganda	T	2	1	5	2	3	3	2	3	2	2	4	4	2	2	5	1	5	3	3	4	2	7	2	2
1657/03	Germany	URAL1	2	4	4	2	2	9	2	3	2	2	4	4	4	2	5	1	1	3	3	3	3	8	3	2
17	Sierra Leone	LAM	2	5	4	2	1	4	2	4	2	4	2	4	1	1	6	1	4	3	5	3	2	9	2	2
1797/03	Germany	EAI	2	2	4	5	3	4	3	6	2	3	6	3	1	1	6	2	2	3	3	6	6	6	1	4
18	Sierra Leone	X	2	2	3	2	6	4	3	4	2	3	3	4	4	2	5	1	5	3	3	3	3	7	3	2
1805/02	Germany	CAS	2	4	2	2	3	4	4	2	2	2	4	4	2	2	5	1	3	3	3	5	3	5	4	3
1850/03	Germany	LAM	2	4	4	2	1	4	2	3	2	2	2	4	1	1	6	1	4	3	5	3	3	3	2	2
1897/04	Germany	S	3	3	5	3	4	3	3	1	2	3	3	4	2	2	5	1	5	4	2	3	3	2	2	2
2111/99	Uganda	T	2	1	5	2	3	3	2	3	2	3	4	4	2	2	5	1	5	3	3	4	2	4	2	2
2151/03	Germany	S	2	3	4	3	4	3	3	1	2	2	4	4	2	2	5	1	5	3	3	3	7	7	2	2
2173/99	Uganda	T	2	1	5	2	2	3	2	3	2	2	4	4	2	2	5	1	4	3	3	4	2	3	2	2
2191/99	Uganda	T	2	2	5	2	3	3	3	3	1	2	4	4	2	1	5	1	4	3	3	4	4	7	2	2
2197/99	Uganda	T	2	2	5	2	3	3	3	3	1	2	3	4	2	1	5	1	4	3	3	4	3	3	2	2
2224/99	Uganda	T	2	3	5	2	2	3	3	2	2	3	4	4	2	2	5	1	5	3	4	5	3	6	2	2
2258/03	Germany	TUR	2	4	4	1	2	5	1	3	2	2	3	4	4	2	5	1	1	3	3	3	3	7	3	2
2263/99	Uganda	T	2	1	5	2	3	3	2	3	2	3	4	4	2	2	5	1	4	3	3	4	2	4	2	2
2307/99	Uganda	T	2	2	5	2	3	3	3	3	1	2	4	4	2	1	5	1	4	4	3	4	3	6	2	2
2318/06	Germany	S	3	4	4	3	5	2	3	1	2	5	3	2	2	2	5	1	5	3	3	3	3	8	2	2
2319/99	Uganda	T	2	2	5	2	3	3	3	3	1	2	4	4	2	1	5	1	4	3	3	4	3	7	2	2
2336/02	Germany	Haarlem	2	1	3	2	3	5	3	3	2	3	3	4	4	2	5	1	5	3	3	3	3	5	3	2
2379/99	Uganda	T	2	2	5	2	3	3	3	3	1	2	3	4	2	1	5	1	4	3	3	4	3	7	2	2
24	Sierra Leone	LAM	1	3	4	2	5	6	3	3	2	2	2	4	1	2	6	1	4	3	1	2	2	5	2	2
2570/02	Ghana	T	2	3	3	2	5	3	3	4	2	3	3	4	4	2	5	1	4	3	2	3	3	6	1	3
2582/02	Ghana	T	2	2	3	2	5	3	3	4	2	4	3	4	4	2	5	1	4	3	2	3	3	6	1	3
2597/02	Ghana	T	2	2	3	2	5	3	3	4	2	3	3	4	4	2	5	1	4	3	2	3	3	6	1	3
26	Sierra Leone	LAM	1	3	4	2	2	4	3	3	2	2	2	4	1	2	6	1	6	3	3	3	2	7	3	2
2637/02	Germany	CAS	2	5	2	2	3	5	5	4	2	2	4	4	2	2	6	1	7	3	3	5	2	8	4	2
2679/03	Germany	URAL1	2	3	4	3	2	9	2	3	2	1	5	4	4	2	5	1	1	3	3	3	3	8	3	2
27	Sierra Leone	LAM	1	3	4	2	7	5	3	3	2	2	2	4	1	2	6	1	5	3	3	2	2	5	2	2
28	Sierra Leone	Cameroon	2	2	6	2	3	3	2	3	1	3	3	4	2	2	5	1	5	3	3	3	10	5	2	2
282/04	Germany	S	2	3	4	3	6	3	3	1	2	5	4	4	2	2	5	1	3	3	3	3	3	5	0	2
3	Sierra Leone	LAM	1	3	2	2	7	6	3	3	2	2	6	4	1	2	6	1	5	3	3	2	2	5	2	2
30	Sierra Leone	LAM	2	4	4	2	1	2	1	3	1	4	2	4	1	1	6	1	4	1	5	3	2	3	2	2
3103/03	Germany	Haarlem	2	2	3	2	3	5	3	3	2	5	3	2	4	2	3	1	5	3	3	3	3	7	3	2
32	Sierra Leone	Haarlem	2	1	3	2	2	5	3	2	2	6	3	4	4	2	5	1	5	3	3	3	3	5	3	2
3243/02	ner Soviet U	Beijing	2	4	4	2	3	3	3	5	2	3	4	4	4	2	5	1	7	3	3	5	3	7	2	3
3256/02	ner Soviet U	Beijing	2	4	4	2	3	3	3	5	2	6	4	4	4	2	5	1	5	3	3	5	3	8	2	3
3262/02	ner Soviet U	LAM	1	3	2	2	4	4	3	3	2	2	2	4	1	2	5	1	5	3	3	2	2	6	2	2
3270/04	Germany	S	2	2	4	3	6	3	2	1	2	2	4	4	2	2	5	1	5	3	2	3	1	9	2	2
3277/02	ner Soviet U	Beijing	2	4	4	2	3	3	3	5	2	6	4	4	4	2	5	1	5	3	3	5	3	8	2	3
33	Sierra Leone	T	2	2	5	2	4	3	1	2	2	2	2	4	2	2	5	1	5	3	3	3	5	5	2	2
3309/02	ner Soviet U	Beijing	2	4	4	2	3	3	3	5	2	6	4	4	4	2	5	1	5	3	3	5	3	8	2	4
3310/02	ner Soviet U	LAM	1	4	2	2	5	4	3	3	2	2	2	4	1	2	5	1	4	3	3	2	2	6	2	2
3329/02	ner Soviet U	Beijing	2	4	4	2	3	3	3	5	2	6	4	4	4	2	5	1	5	3	3	5	3	7	2	3
3342/02	ner Soviet U	Haarlem	2	2	3	2	3	5	3	1	2	3	3	4	4	2	5	1	5	1	3	3	3	6	3	2
3364/02	ner Soviet U	Beijing	2	4	4	2	3	3	3	5	2	6	4	4	4	2	5	1	5	3	3	5	3	8	2	3
34	Sierra Leone	T	2	2	5	2	4	3	1	2	2	4	3	4	2	2	5	1	5	3	3	3	5	5	2	2
36	Sierra Leone	Haarlem	2	1	3	2	3	5	3	3	2	3	3	4	4	2	5	1	5	3	3	3	3	7	3	2
3686/03	Germany	Haarlem	2	2	3	2	2	4	3	3	2	4	3	4	4	2	5	1	5	3	3	3	4	6	3	2
38	Sierra Leone	Haarlem	2	3	3	2	3	5	3	3	2	3	5	4	4	2	5	1	5	3	3	3	3	5	3	2
39	Sierra Leone	Cameroon	2	2	3	2	3	3	2	3	1	4	3	4	2	2	5	1	5	3	3	3	10	4	2	2
3995/03	Germany	LAM	2	3	2	2	4	4	3	3	2	3	2	4	2	2	6	1	5	3	1	3	1	4	2	2
4	Sierra Leone	Haarlem	2	2	3	2	3	5	3	3	2	3	3	4	4	2	5	1	5	3	3	3	3	5	3	2
40	Sierra Leone	Haarlem	2	3	3	2	5	5	3	4	1	6	5	4	4	2	5	1	4	3	3	3	2	6	1	2
4058/03	Germany	EAI	2	2	4	5	4	4	3	6	2	3	6	3	1	1	4	2	2	3	3	4	4	5	1	3
41	Sierra Leone	Beijing	3	3	5	2	3	3	3	4	2	6	3	4	4	2	6	1	6	3	3	5	3	7	2	5
4130/02	Germany	Haarlem	2	2	3	2	3	5	3	3	2	4	3	4	4	2	5	1	5	3	3	3	3	5	3	2
4192/03	Germany	Haarlem	2	2	3	2	3	2	3	3	2	6	3	4	4	2	5	1	5	3	3	3	3	6	3	2
4217/02	Germany	Haarlem	2	2	3	2	2	4	3	3	2	6	3	4	4	2	2	1	5	3	3	3	4	7	3	2
44	Sierra Leone	Haarlem	2	3	3	2	3	5	3	3	2	3	5	4	4	2	5	1	5	3	3	3	3	5	3	2
4412/04	Germany	X	2	2	3	2	5	4	3	4	2	4	3	4	4	2	5	1	5	3	3	3	6	8	3	2
4428/02	ner Soviet U	LAM	2	3	4	2	6	4	3	3	2	1	1	4	1	2	7	1	5	3	3	2	2	6	2	2
4431/02	ner Soviet U	LAM	1	3	2	2	5	4	3	3	2	2	2	4	1	2	5	1	4	3	3	2	2	5	2	2
4436/02	ner Soviet U	Beijing	2	4	4	2	3	3	3	5	2	6	4	4	4	2	5	1	7	3	3	5	3	7	2	3
4445/02	ner Soviet U	Beijing	2	4	4	2	3	3	3	5	2	6	3	3	4	2	5	1	5	3	3	5	3	8	2	3
4498/02	ner Soviet U	Beijing	2	4	4	2	3	3	3	6	2	6	4	4	4	2	5	1	5	3	3	5	3	8	2	3
4499/02	ner Soviet U	Beijing	2	4	4	2	3	3	3	3	2	6	4	4	4	2	5	1	5	3	3	5	3	8	2	3
45	Sierra Leone	T	2	4	3	2	2	3	4	2	2	3	3	4	2	2	5	1	4	3	3	3	2	7	2	2
4526/04	Germany	S	3	3	4	3	5	3	2	1	2	3	4	4	2	2	5	1	4	3	2	3	3	8	2	2
46	Sierra Leone	S	2	3	3	3	2	3	3	1	2	5	3	4	2	2	5	1	3	3	3	3	2	4	1	2
48	Sierra Leone	LAM	1	3	3	2	2	3	3	3	2	2	3	4	1	2	6	1	5	3	3	3	2	8	3	2
4850/03	Germany	EAI	2	2	4	5	4	3	3	6	2	3	6	3	1	1	7	2	2	3	3	3	4	5	1	3
49	Sierra Leone	Haarlem	2	3	3	2	3	5	3	3	2	3	5	4	4	2	5	1	5	3	3	3	3	5	3	2
4993/02	Germany	Haarlem	2	3	3	2	3	5	3	3	2	4	3	4	4	2	5	1	5	3	3	3	3	5	3	2
50	Sierra Leone	T	2	1	3	2	4	3	1	2	2	3	3	4	2	2	5	1	5	3	3	3	6	5	2	2
51	Sierra Leone	T	2	1	3	2	4	3	1	2	2	3	3	4	2	2	5	1	5	3	3	3	6	5	2	2
53	Sierra Leone	LAM	1	3	2	2	7	6	3	3	2	2	6	4	1	2	6	1	5	3	3	2	2	5	2	2
5357/02	Ghana	Ghana	3	2	2	3	2	7	3	3	4	2	4	3	4	5	2	5	1	4	3	2	3	3	6	1
5390/02	Ghana	Cameroon	2	2	4	2	3	3	2	3	1	6	3	4	2	2	5	1	5	3	3	3	8	5	3	2
54	Sierra Leone	Haarlem	2	2	3	2	3	5	3	3	2	3	3	4	4	2	5	1	5	3	3	3	3	5	3	2
5400/02	Ghana	Cameroon	2	2	4	2	3	4	3	3	1	5	3	4	2	2	5	1	5	3	3	3	7	5	2	2
5429/02	Ghana	Cameroon	2	2	4	2	3	3	3	3	1	5	4	4	2	2	5	1	5	3	3	3	a	5	2	2
56	Sierra Leone	T	2	2	5	2	4	3	1	2	2	3	2	4	2	2	5	1	5	3	3	3	5	5	2	2
58	Sierra Leone	EAI	2	2	4	3	3	6	2	6	2	9	7	3	2	6	5	2	2	4	3	5	10	6	1	3

60	Sierra Leone	Haarlem	2	2	3	2	3	5	3	3	2	3	3	4	4	2	5	1	5	3	3	3	3	5	3	2
6006/03	Germany	EAI	2	2	2	4	3	4	3	3	2	8	8	3	2	4	6	1	2	3	3	4	5	4	1	1
61	Sierra Leone	T	2	2	25	2	2	3	4	2	2	5	2	4	2	1	5	1	5	3	3	3	3	5	2	2
62	Sierra Leone	Cameroon	2	2	6	2	3	3	2	3	1	4	3	4	2	2	5	1	5	3	3	3	10	4	2	2
6411/05	Germany	S	3	3	4	3	5	3	3	1	2	4	3	4	2	2	5	1	5	4	2	3	4	4	0	2
6424/05	Germany	S	3	3	4	3	4	3	3	1	2	3	4	4	2	2	5	1	4	3	2	3	3	8	2	2
6427/01	Germany	CAS	2	5	2	2	3	5	4	4	2	2	4	4	2	2	6	1	7	3	3	5	2	8	4	2
6538/03	Germany	EAI	2	2	4	5	3	4	3	4	2	3	6	3	1	4	6	2	2	3	3	5	4	6	1	3
66	Sierra Leone	S	2	3	4	3	2	3	3	1	2	5	4	4	2	2	5	1	3	3	3	3	2	4	1	2
67	Sierra Leone	LAM	1	3	4	2	4	2	3	3	2	2	2	4	1	2	6	1	5	3	3	2	2	7	2	2
68	Sierra Leone	Haarlem	2	2	3	2	3	5	3	3	2	3	3	4	4	2	5	1	5	3	3	3	3	5	3	2
6946/03	Germany	Haarlem	2	2	3	2	2	4	3	3	2	6	3	4	4	2	2	1	5	3	3	3	4	7	3	2
7	Sierra Leone	LAM	1	3	4	2	2	3	3	3	2	2	2	4	1	2	6	1	5	3	3	3	2	8	3	2
70	Sierra Leone	S	2	3	4	3	4	2	3	1	2	4	3	4	2	2	5	1	5	3	2	3	3	5	2	2
71	Sierra Leone	EAI	2	2	4	3	3	6	3	6	2	11	7	3	2	6	5	2	2	4	3	5	2	6	1	3
7190/03	Germany	EAI	2	2	4	5	3	4	4	6	2	9	6	3	2	1	6	2	2	3	3	5	5	6	1	3
72	Sierra Leone	T	2	2	4	2	4	3	1	2	2	2	3	4	2	2	5	1	5	3	3	3	5	5	2	2
73	Sierra Leone	Haarlem	2	1	3	2	3	5	3	3	2	3	3	4	4	2	5	1	5	3	3	3	3	7	3	2
74	Sierra Leone	Cameroon	2	2	4	2	5	3	3	3	1	4	2	4	2	2	5	1	5	3	4	3	7	5	2	2
742/06	Germany	S	3	4	4	3	5	2	3	1	2	5	3	2	2	2	5	1	5	3	3	3	3	8	2	2
75	Sierra Leone	Haarlem	2	2	3	2	3	5	2	3	2	4	3	4	4	2	5	1	5	3	3	3	3	5	3	2
7507/01	Germany	CAS	2	4	2	2	3	6	4	5	2	2	4	4	2	2	5	1	9	3	3	4	3	8	4	3
76	Sierra Leone	T	2	2	3	2	1	4	3	4	2	3	3	4	4	2	5	1	5	3	3	3	3	7	3	2
7746/01	Germany	CAS	2	3	2	2	3	6	2	4	2	2	4	4	2	2	5	1	7	3	3	5	3	2	2	3
7747/01	Germany	CAS	2	5	2	2	3	5	4	4	2	2	4	4	2	2	6	1	4	3	3	5	2	7	4	2
78	Sierra Leone	T	2	2	5	2	4	3	1	2	2	2	2	4	2	2	5	1	5	3	3	3	5	5	2	2
79	Sierra Leone	T	2	2	2	2	4	3	1	2	2	3	3	4	2	2	5	1	5	3	2	3	6	5	2	2
7955/03	Germany	S	2	3	4	3	4	3	2	1	2	4	4	4	2	2	5	1	5	3	3	3	3	a	2	2
7968/03	Germany	LAM	2	3	2	2	3	4	3	3	2	4	2	4	2	2	6	1	5	3	1	3	1	4	2	2
8	Sierra Leone	Beijing	2	4	4	2	3	3	3	4	2	4	2	4	4	2	5	1	6	3	3	5	3	7	2	3
80	Sierra Leone	Haarlem	2	2	3	2	3	4	3	3	2	3	3	4	4	2	5	1	5	3	3	3	3	5	3	2
82	Sierra Leone	Haarlem	2	2	3	2	3	5	3	3	2	3	3	4	4	2	5	1	5	3	3	3	3	5	3	2
8260/01	Germany	CAS	2	4	2	2	3	5	4	4	2	2	4	4	2	2	4	1	8	3	3	4	3	8	4	3
83	Sierra Leone	T	2	2	5	2	4	3	1	2	2	3	2	4	2	2	5	1	5	3	3	3	5	5	2	2
84	Sierra Leone	EAI	2	2	4	5	2	6	3	6	2	9	7	3	2	6	5	2	3	4	3	5	10	3	1	1
8431/03	Germany	URAL1	2	2	4	2	2	a	2	5	2	2	4	4	4	2	5	1	1	3	3	2	3	8	3	2
85	Sierra Leone	LAM	2	4	4	2	1	2	1	3	2	4	2	4	1	1	6	1	4	2	5	3	2	3	2	2
8577/03	Germany	URAL1	2	3	5	2	3	7	2	3	2	2	4	4	4	2	5	1	1	3	3	2	3	6	3	2
86	Sierra Leone	Haarlem	2	2	3	2	4	5	3	3	1	5	3	2	4	2	3	1	5	3	3	3	3	7	3	2
8750/03	Germany	Haarlem	2	2	4	2	2	6	3	3	2	4	2	4	4	2	5	1	5	3	3	3	3	5	3	2
8870/03	Germany	NEW-1	2	2	4	2	3	2	2	5	2	2	3	4	2	1	5	1	4	3	2	3	3	7	2	2
8885/03	Germany	LAM	2	4	4	2	1	3	2	3	2	3	2	4	1	1	6	1	4	1	5	3	2	8	2	2
8915/03	Germany	EAI	2	1	4	5	2	4	3	a	2	8	4	3	2	6	6	2	2	3	3	4	2	7	1	3
9	Sierra Leone	Beijing	2	4	4	2	3	3	3	4	2	4	2	4	4	2	5	1	6	3	3	5	3	7	2	3
91	Sierra Leone	EAI	2	2	4	3	3	5	5	4	2	8	7	3	2	4	5	2	2	3	3	4	7	6	1	3
9267/01	Germany	EAI	2	1	4	5	2	4	3	a	2	9	4	3	2	6	6	2	2	3	3	4	2	7	1	3
93	Sierra Leone	Beijing	2	4	4	2	3	3	3	4	2	6	5	4	4	2	5	1	6	3	3	5	3	7	2	3
9398/01	Germany	CAS	2	5	2	2	3	5	4	4	2	2	4	4	2	2	6	1	7	3	3	5	2	8	4	2
94	Sierra Leone	LAM	2	4	4	2	1	4	2	3	2	3	2	4	1	2	6	1	5	3	3	3	2	8	2	1
9400/02	Germany	Haarlem	2	2	3	2	4	5	3	3	2	6	3	2	4	2	4	1	5	3	3	3	3	7	3	2
946/03	Germany	LAM	2	1	4	2	1	3	1	3	2	2	2	5	1	2	6	1	5	3	3	3	3	4	2	2
947/01	Germany	EAI	2	2	4	4	2	4	3	4	2	8	a	3	2	4	7	2	2	1	3	6	4	6	1	3
95	Sierra Leone	LAM	2	2	4	2	1	4	2	3	2	3	2	4	1	2	6	1	5	3	3	3	2	8	2	2
9532/03	Germany	Haarlem	2	2	3	2	3	5	3	3	2	3	3	4	4	2	5	1	5	3	3	3	3	3	3	2
96	Sierra Leone	T	2	2	3	2	2	4	3	4	2	3	3	4	4	2	5	1	5	3	3	3	3	7	3	2
97	Sierra Leone	LAM	1	3	4	2	7	6	3	3	2	2	2	4	1	2	6	1	5	3	3	2	2	5	2	2
9787/04	Germany	X	2	2	3	2	5	4	3	4	2	4	3	4	4	2	5	1	5	3	3	3	6	8	3	2
9915/01	Germany	CAS	2	5	2	2	3	5	4	4	2	2	4	4	2	2	6	1	7	3	3	5	2	8	4	2
ET101	Eastern Afric	CAS	2	2	2	2	1	7	4	3	2	2	4	4	2	2	5	1	4	3	3	4	2	5	4	3
ET102	Eastern Afric	CAS	2	3	2	2	3	7	4	4	2	2	4	4	4	2	5	1	3	3	3	4	3	9	4	2
ET103	Eastern Afric	CAS	2	4	2	2	5	6	4	2	2	2	4	3	2	2	5	1	3	3	3	5	3	7	3	3
ET104	Eastern Afric	CAS	2	3	2	2	3	6	4	4	2	2	4	4	2	2	5	1	5	3	3	5	3	7	7	3
ET106	Eastern Afric	CAS	2	3	2	2	5	6	4	4	2	2	3	4	2	2	5	1	3	3	3	5	3	8	4	3
ET115	Eastern Afric	CAS	2	4	2	2	7	4	3	4	2	2	4	4	2	2	5	1	3	3	3	5	3	7	4	3
ET119	Eastern Afric	CAS	2	3	2	2	5	6	4	4	2	2	4	4	2	2	5	1	3	3	3	5	3	7	4	3
ET12	Eastern Afric	CAS	2	3	2	2	5	5	4	4	2	2	4	4	2	2	5	1	3	3	3	5	3	7	4	3
ET122	Eastern Afric	CAS	2	4	2	2	5	6	4	2	2	2	4	4	2	2	5	1	3	3	3	5	2	7	3	3
ET125	Eastern Afric	CAS	2	3	2	2	3	6	4	4	2	2	4	4	2	2	5	1	5	3	3	5	3	4	4	3
ET130	Eastern Afric	CAS	2	3	2	2	3	5	4	4	2	2	4	4	2	2	5	1	5	3	3	5	3	7	4	3
ET132	Eastern Afric	CAS	2	3	2	2	3	6	4	4	2	2	4	4	2	2	6	1	4	3	3	5	3	7	4	3
ET135	Eastern Afric	CAS	2	1	2	2	3	6	4	4	2	2	4	4	2	2	5	1	5	3	3	6	3	7	4	3
ET14	Eastern Afric	CAS	2	4	2	2	1	7	4	3	2	2	4	4	2	2	5	1	4	3	3	4	2	4	4	3
ET141	Eastern Afric	CAS	2	3	2	2	3	6	4	4	2	2	0	4	2	2	5	1	5	3	3	5	3	8	4	3
ET146	Eastern Afric	CAS	2	1	2	2	3	6	4	4	2	2	4	4	2	2	5	1	5	3	3	5	3	7	4	3
ET153	Eastern Afric	CAS	2	3	2	2	3	6	4	4	2	2	4	4	2	2	5	2	5	3	3	5	3	7	4	3
ET16	Eastern Afric	CAS	2	3	2	2	5	6	4	4	2	2	4	4	2	2	5	1	4	3	3	5	3	7	4	3
ET165	Eastern Afric	CAS	2	3	2	2	3	6	4	4	2	2	0	4	2	2	5	1	5	3	3	5	3	8	4	3
ET167	Eastern Afric	CAS	2	4	2	2	4	0	4	3	2	2	4	4	2	2	5	1	1	3	3	4	2	5	4	3
ET173	Eastern Afric	CAS	2	1	2	2	3	6	4	4	2	2	4	4	2	2	5	1	5	3	3	6	3	7	4	3
ET178	Eastern Afric	CAS	2	4	2	2	3	4	3	4	2	2	4	4	2	2	5	1	7	3	3	5	3	8	4	3
ET18	Eastern Afric	CAS	2	3	2	2	5	6	4	2	2	2	4	4	2	2	5	1	3	3	3	5	3	7	4	3
ET19	Eastern Afric	CAS	2	1	2	2	5	6	4	4	2	2	4	4	2	2	5	1	3	3	3	6	3	7	4	3
ET190	Eastern Afric	CAS	2	3	2	2	3	6	4	4	2	2	0	4	2	2	5	1	5	3	3	5	5	8	4	3
ET194	Eastern Afric	CAS	2	3	2	2	3	4	4	4	2	2	4	4	2	2	5	1	5	3	3	5	3	4	4	3
ET198	Eastern Afric	CAS	2	4	2	2	3	4	3	4	2	2	4	4	2	2	5	1	7	3	3	5	3	7	4	3
ET20	Eastern Afric	CAS	2	3	2	2	5	6	4	4	2	2	4	4	2	2	5	1	3	3	3	5	3	7	4	3
ET201	Eastern Afric	CAS	2	1	2	2	3	5	4	4	1	2	4	4	2	2	5	1	5	3	3	6	3	7	4	3
ET204	Eastern Afric	CAS	2	3	2	2	3	6	4	4	2	2	0	4	2	2	5	1	5	3	3	5	5	8	4	3
ET206	Eastern Afric	CAS	2	3	2	2	3	6	3	4	2	2	4	2	2	2	5	1	4	3	3	5	3	7	4	3
ET207	Eastern Afric	CAS	2	3	2	2	3	6	4	3	2	2	6	4	2	2	5	1	5	3	3	5	3	8	4	3
ET208	Eastern Afric	CAS	2	5	2	2	3	4	4	4	2	2	4	4	2	2	5	1	7	3	3	5	3	8	2	3
ET210	Eastern Afric	CAS	2	3	2	2	3	6	4	4	2	2	4	4	2	2	5	1	5	3	3	5	3	7	4	3
ET217	Eastern Afric	CAS	2	1	2	2	3	6	4	4	2	2	4	4	2	2	5	1	5	3	3	6	3	7	4	3
ET219	Eastern Afric	CAS	2	4	2	2	3	4	3	4	2	2	4	4	2	2	5	1	7	3	3	5	3	7	4	3
ET222	Eastern Afric	CAS	2	3	2	2	3	6	4	4	2	2	4	4	2	2	6	1	4	3	3	5	3	7	4	3
ET23	Eastern Afric	CAS	2	3	2	2	5	6	4	3	2	2	0	4	2	2	5	1	3	3	3	5	3	8	5	3
ET230	Eastern Afric	CAS	2	3	2	2	3	6	5	4	2	2	4	4	2	2	5	1	5	3	4	5	3	5	4	3
ET231	Eastern Afric	CAS	2	4	2	2	3	6	4	4	2	2	0	4	2	2	5	1	5	3	3	5	3	8	4	3
ET24	Eastern Afric	CAS	2	3	2	2	5	4	4	2	2	2	6	4	2	2	5	1	3	3	3	5	3	8	4	3
ET241	Eastern Afric	CAS	2	4	2	2	3	4	3	4	2	2	4	4	2	2	5	1	7	3	3	5	3	7	4	3
ET242	Eastern Afric	CAS	2	4	2	2	3	6	4	2	2	2	4	4	2	2	5	1	5	3	3	5	1	7	3	3
ET244	Eastern Afric	CAS	2	3	2	2	2	7	4	6	2	2	4	4	2	2	5	1	6	3	3	5	2	6	4	3
ET245	Eastern Afric	CAS	2	3	2	2	3	6	4	4	2	2	4	4	2	2	5	1	5	3	3	5	3	7	4	3
ET250	Eastern Afric	CAS	2	1	2	2	3	6	3	4	2	2	4	4	2	2	5	1	5	3	3	6	3	7	4	3
ET257	Eastern Afric	CAS	2	1	2	2	3	6	4	4	2	2	4	4	2	2	5	1	5	3	3	6	3	7	4	3
ET260	Eastern Afric	CAS	2	4	2	2	3	4	3	4	2	2	4	4	2	2	5	1	7	3	2	5	3	7	4	3
ET263	Eastern Afric	CAS	2	3	2	2	3	6	5	4	2	2	4	3	2	2	5	1	5	3	3	5	3	7	4	3
ET264	Eastern Afric	CAS	2	4	2	2	3	4	3	4	2	2	4	4	2	2	5	1	7	3	2	5	3	7	4	3
ET266	Eastern Afric	CAS	2	3	2	2	3	6	4	4	2	2	4	4	2	2	5	1	5	3	3	5	3	7	4	3
ET269	Eastern Afric	CAS	2	1	2	2	3	6	4	4	2	2	4	4	2	2	5	1	5	3	3	6	3	7	4	3
ET276	Eastern Afric	CAS	2	1	2	2	3	6	4	4	2	2	4	4	2	2	5	1	5	3	3	6	3	7	4	3
ET278	Eastern Afric	CAS	2	4	2	2	4	7	4	3	2	0	4	4	2	2	5	1	1	3	3	4	2	5	4	3
ET28	Eastern Afric	CAS	2	3	2	2	4	6	4	2	2	2	6	4	2	2	5	1	3	3	3	5	3	8	4	3
