# Supplementary material for: The shortcut of mycobacterial interspersed repetitive unit-variable number tandem repeat typing for Mycobacterium tuberculosis differentiation
Source: Front Microbiol. 2022 Sep 8;13:978355. doi: 10.3389/fmicb.2022.978355 (PMC9493315; doi:10.3389/fmicb.2022.978355)
Supplement: Supplementary file 1 [file Data_Sheet_1.PDF]

Supplementary Table S1: MIRU-VNTR and Spoligotype patterns of all studied Mtb isolates.

ID	Genotype	154	424	577	580	802	960	1644	1955	2059	2163b	2165	2347	2401	2461	2531	2687	2996	3007	3171	3192	3690	4052	4156	4348
1	Haarlem	2	4	2	3	3	5	3	6		2	4	4	4	2	5	1	5	3	3	3	4	8		2
2	NEW1	2	1	5	2	2	2	2	4		2	3	4	2	1	5	1	5	3	2	3	3	3		1
3	CAS-Delhi	2	5	2	2	2	5	3	4	2			4	2	2	5	1	7	3	3	3	3	8		2
4	Beijing	2	4	4	2	2	3	2	5	2	6	4	4	4	2	5	1	5	3	3	3	3	8		2
5	Haarlem	2	1	2	2	2	5	1	3	2	2	3	4	4	2	5	1	1	3	3	3	3	8		2
6	Beijing	2	4	4	2	3	3	2	5	2	6	4	3	4	2	5	1	5	1	3	5	3	8	2	3
7	Haarlem	2	1	2	2	2	5	1	3	2	2	3	4	4	2	5	1	1	3	3	3	3	8	2	2
8	NEW1	2	2	4	2	3	2	2	5	2	2	3	4	2	1	5	1	5	3	2	3	3	7	2	2
9	CAS-Delhi	3	2	2	2	3	6	3	6	3	1	3	4	2	2	5	1	5	3	3	5	3	6	2	3
10	NEW1	3	2	4	2	3	2	2	5		2	2	4	2	1	5	1	5	3	2	3	3	7	2	2
11	CAS-Delhi	3	5	2	2	3	5	3	4			3	4	2	2	5	1	7	3	3	5	3	9		3
12	Beijing	3	4	4	2	3	3	1	5		6	4	4	4	2	5	1	5	1	3	5		9	2	3
13	Haarlem	3		4	3	2	5	1	3		2	3	4	2	2	5	1	1	3	3	3	3	9		2
14	CAS-Delhi	2		2	3	3	5	4	4		2	3	4	2	2	5	1	7	3	2	5	3	6	4	4
15	NEW1	2	5	4	3	3	2	3	3		2	3	4	2	1	5	1	5	3	3	3	3	12	2	2
16	Ural1	2		4	3	2	9	2	3		2	3	4	5	2	5	1	1	3	3	3	3	10	2	2
17	NEW1	2		4	2	3	2	3	3	3	4	3	4	2	1	5	1	5	3	2	3	3	6	2	3
18	NEW1	2	2	4	2	4	2	3	5		2	3	4	2	1	5	1	5	3	2	3	3	7	2	2
19	Beijing	2		4	2	3	3	3	5		6	4	4	4	2	5	1	5	3	3	4	3	9		4
20	Beijing	5	5	4	2		3	3	5		6	4	4	4	2	5	1	5	3	3	4		9		4
21	NEW1	2	2	4	2	3	2	3	5		2	3	4	2	1	5	1	5	3	2	4	4	7	2	3
22	CAS-Delhi	3	3	2	2	3	6	6	6		2	3	4	2	2	5	1	5	3	3	5	4	6	2	4
23	Beijing	2	3	4	2	3	4	3	5		6	4	4	5	2	5	1	5	1	3	5	3	9	2	3
24	Haarlem	2	2	4	2	3	3	4	3		3	3	4	3	1	5	1	6	3	3	3	3	7	2	2
25	Haarlem	2	2	4	2	6	5	2	2			3	4	2	2	5	1	6	3	3	5	5	6	2	2
26	CAS-Delhi	4	4	2	2	3	7	4	6		2	3	3	2	2	5	1	5	3	3	3	3	9	2	3
27	Haarlem	2	2	3	2	3	6	4	3		3	3	3	4	2	5	1	5	3	3	3	3	5	2	2
28	Ural1	5	5	4	2	1	6	2	3	3	3	3	3	4	2	5	1	1	3	2	3	3	10	3	2
29	Ural1	4	4	4	2	0	7	3	3		2	4	3	5	2	5	1	1	3	3	1	2	4	3	2
30	Haarlem	2	2	4	2	4	5	3	2		4	3	4	2	2	5	1	3	2	3	3		5	2	2
31	CAS-Delhi	5	5	2	2	3	6	4	3	3	2	4	4	2	2	5	1	7	3	3	3	2	8	3	4
32	NEW1	2	2	4	2	3	2	2	5	3	2	3	4	2	1	5	1	6	3	3	3	3	8	2	2
33	Cameroon	1	2	4	2	3	3	2	2		6	3	4	2	2	6	1	5	3	3	3	3	5	2	2
34	NEW1	2	2	4	2	3	2	2	2		2	3	4	2	1	5	1	6	3	3	3	3	5	2	2
35	NEW1	2	1	5	2	3	2	2	3		2	3	4	2	1	5	1	6		2	3	3	3	2	
36	NEW1	2	2	4	2	3	2	2	5		2	3	4	2	1	5	1	5	3	2	3	3	3		
37	Ural1	2	3	5	2	3	9	3	3	3	2	4	4	4	2	5	1	1	3	3	2	3	5		3
38	Beijing	2	5	4	2	3	3	3	5		6	4	4	4	2	5	1	5	3	3	5	4	9		3
39	CAS-Delhi	2	6	2	2	3	6	4	4	3	2		4	2	2	5	1	7	3	3	5	4	9		3
40	NEW1	2	2	4	2	3	2	3	5		2	3	4	2	1	5	1	6	3	2	3	4	7	2	2
41	NEW1	2	2	4	2	3	2	2	4	3	2	3	4	2	1	5	1	5	3	2	5	3	8		2
42	Beijing	2	2	5	2	2	3	3	4	3	3	4	4	2	2	5		7	3	3	5	4	9	4	
43	NEW1	2	2	4	2	3	2	3	5	3	2	3	4	2	1	5	1	6	3	2	3	3	8		
44	CAS-Delhi	2	5	2	2	3	5	4	4	3	2	4	4	2	2	5	1	7	3	3	5	3	8		4
45	CAS-Delhi	2	5	2	2	3	7	4	4	3	2	2	4	5	2	5	1	10	3	3	5	3	4		4
46	CAS-Delhi	2	5	2	2	3	5	4	3	3	2	4	4	5	2	5	1	10	3	3	5	3	4		3
47	CAS-Delhi	2	5	2	2	3	6	4	4	3	2	4	4	2	2	5	1	2	3	3	5	3	7		3
48	Beijing	2	5	4	2	3	3	3	5	2	6	4	4	5	2	5	1	5	3	3	4	3	6	2	4
49	NEW1	2	2	4	2	3	2	3	5	2	2	3	4	2	1	5	1	5	3	2	3	4	7	0	2
50	CAS-Delhi	2	5	2	2	3	5	5	3	2	2	4	4	5	2	2	1	5	3	3	3	4	8	0	4
51	Beijing	2	5	3	2	3	3	2	5		6	4	4	5	2	5	1	5	1	3	5	3	9	0	4
52	Beijing	2	5	4	2	3	3	3	4		5	4	4	5	2	5	1	5	3	3	5	3	9	0	4
53	CAS-Delhi	2	5	2	2	3	5	5	3		2	4	4	5	2	2	1	5	3	3	3	4	9	0	4
54	Haarlem	2	2	4	2		3	1	2		4	2	4	4	2	5	1	6	3	3	3	3	5	2	4
55	Cameroon	1	3	4	2	3	3	3	2		5	4	4	2	2	2	1	5	3	3	3	3	6		2
56	CAS-Delhi	2	7	2	2	3	5	4	4		2	4	4	2	2	5	1	7	3	3	5	3	5		4
57	Uganda	2	1	4	2	2	3	3	3		2	4	4	2	2	3	1	5	3	2	3	4	3	2	2
58	NEW1	2	2	4	2	3	2	3	5	2	2	2	4	2	1	5	1	6	3	2	3	3	7	2	2
59	NEW1	2	1	4	2	3	2	3	5		2	3	4	2	1	5	1	5	3	2	3	4	5	2	2
60	NEW1	2	3	4	2	4	2	3	5		2	3	4	2	1	5	1	6	3	2	3	3	7	4	2
61	CAS-Delhi	2	4	2	2	3	7	4	4		2	2	4	4	2	5	1	9	3	3	5	3	4	2	4
62	Ural1	2	4	4	2	2	7	2	3		2	4	4	5	2	5	1	3	2	3	3	2	8	4	2
63	CAS-Delhi	2	3	2	2	3	6	4	6			3	4	2	2	5	1	6	3	3	5	3	8		4
64	CAS-Delhi	2	4	2	2	4	6	4	4	2	2	4	4	2	2	5	1	6	3	3	5	3	8	4	4
65	CAS-Delhi	2	2		2	3	7	4	4	2	2	2	4	4	2	5	1	9	3	3	5	3	4	2	3
66	CAS-Delhi	2	2	2	2	4	5	4	4		2	4	4	2	2	5	1	6	2	3	5	3	8	2	4
67	NEW1	2	2	4	2	3	3	2	5		2	3	4	2	1	5	1	6	3	2	3	3	5	2	2
68	NEW1	2	1	4	2	1	3	2	4	2	2	3	4	2	1	5	1		3	2	3	2	8	2	2
69	Haarlem	2	4	3	2	3	7	3	4	2	2	2	4	4	2	5	1	10	3	2	3	3	4	3	2
70	Beijing	2	4	4	2	3	5	3	5	2		4	4	4	2	5	1	5	3	3	5	2	9	2	4
71	NEW1	2	2	4	2	3	2	3	5		2	3	4	2	1	5	1	5	3	2	3	3	6	2	2
72	NEW1	2	2	4	2	3	2	3	3	2	2	2	4	2	1	5	1	6	3	2	3	3	7	2	2
73	CAS-Delhi	2	3	2	2	2	5	4	6		2	2	4	2	2	5	1	5	3	1	5	3	6	5	3
74	Uganda	2	1	5	2	3	3	4	2	2	3	2	4	2	2	6	1	5	3	3	3	3	5	2	2
75	Cameroon	2	1	4	2	2	3	2	2		3	2	4	2	2	6	1	5	3	3	3	4	6	2	2
76	NEW1	2	2	4	2	3	2	3	5		2	3	1	2	1	5	1	6	3	2	3	3	8	2	2
77	Uganda	2	1	4	2	2	3	3	3	2	3	4	4	2	2	3	1	5	3	2	3	5	4	2	2
78	NEW1	2	2	4	2	3	2	3	5		2	3	4	2	1	5	1	6	3	2	3	2	7	2	2
79	NEW1	2	1	4	2	1	2	3	3	1	2	3	4	2	1	5	1	6	3	3	3	3	7	2	2
80	CAS-Delhi	2	5	2	2	3	9	4	4	2	1	4	4	2	2	5	1	7	3	3	3	3	8	3	3
81	NEW1	2	2	4	2	3	2	3	5			3	4	2	1	5	1	5	2	2	4	3	7		2
82	CAS-Delhi	2	4	2	2	3	5	3	6	2	2	3	4	2	2	5	1	5	3	3	4	4	6	0	2
83	NEW1	2	2	4	2	3	2	3	5	2	2	3	4	2	1	5	1	5	2	2	3	3	4	2	2
84	CAS-Delhi	2	5	2	2	3	3		4		2	4	4		2	5	1	8	3	3	5	2	7		
85	NEW1	2	2	4	2	4	2	3	5		2	2	4	2	1	5	1	4	3	2	3	4	6	2	2
86	Haarlem	2	2	3	2	3	5	3	3	2	7	3	4	4	2	5	1	5	3	3	4	3	6		2
87	Beijing	2	3	4	2	3	3	2	5	2	6	4	4	4	2	5	1	5	1	3	5	3	8		3
88	NEW1	2	2	4	2	3	2	3	4	2	2	3	4	2	1	5	1	6	3	2	3	3	7	2	2
89	NEW1	2	3	4	2	3	2	3	2	2	2	2	4	2	1	5	1	5	3	3	3	3	5	2	2
90	Cameroon	2	2	4	2	4	3	3	2	2	2	2	4	2	2	5	1	5	3	3	3	3	5	2	2
91	CAS-Delhi	2	2	2	2	3	5	4	4	2	2	4	4	2	2	5	1	7	3	3	5	3	8	1	3
92	NEW1	2	2	4	2	3	2	3	5	2	2	3	4	2	1	5	1	5	3	2	3	3	7	2	2
93	Cameroon	1	3	4	2	3	3	3	2	2	5	3	4	2	2	2	1	5	3		3	3	4	2	2
94	Ural1	2	4	4	2	2	6	2	3	2	2	4	4	5	2	5	1	1	2	3	3	2		4	2
95	CAS-Delhi	2	5	2	2	3	5	4	4	2		4	4	2	2	5	1	7	3	3	5	3	8	4	4
96	Beijing	2	4	4	2	3	3	2	5	2	6	4	4	4	2	5	1	5	1	3	5	3	8	2	4
97	NEW1	2	2	4	2	3	2	3	4	2	2	3	4	2	1	5	1	5	3	2	3	3	7		
98	CAS-Delhi	2	3	2	2	2	3	5	4	2	2	4	4	4	2	5	1	7	3	3	5	4	3	4	4
99	CAS-Delhi	2	5	2	2	3	5	4	4	2			4	2	2	5	1	7	3	3	5	3	8		4
100	NEW1	2	2	4	2	3	2	3	5	2	2	3	4	2	1	5	1	5	3	2	3	3	7	2	3
101	CAS-Delhi	2	5	2	3	5	6	4	4		2	4	4	2	2	5	1	7	2	2	3	3	8	2	3
102	CAS-Delhi	2	2	1	2	3		4	4	2	2	4	4	2	2	5	1	7	3	3	5	3	8	1	2
103	NEW1	2	2	4	2	3	2	3	5	2	2		4	2	1	5	1	6	3	2	3	3	6	2	2
104	CAS-Delhi	2	4	2	2	3	5	5	4	2			4	4	2	5	1	9	3	3	4	3	3	4	3
105	Beijing	2	4	4	2	2	3	2	4		6	4	4	4	2	5	1	5	1	3	4	3	8	2	
106	NEW1	2	2	4	2	2	2	3	5		2	3	5	2	1	5	1	6		2	3	3	6	2	3
107	NEW1	2	2	4	2	1	2	3	5		2	2		2	1	5	1	6	3	2	3	3	7	2	3
108	NEW1	2	2	4	2	2	2	3	5		2	3	4	2	1	5	1	5	3	2	4	3	7	2	3
109	CAS-Delhi	2	4	2	2	3	6	4	6		2	3	4	2	2	5	1	5	3	3	4	4	6	4	3
110	CAS-Delhi	2	4	2	2	3	6	4	6		2	3	4	2	2	5	1	5	3	3	4	4	6		
111	NEW1	2	2	7	2	3	2	3	5		2	3	4	2	1	5	1	6	3	2	3	3	8		2
112	Beijing	2	3	4	2	3	3	2	5	2	7	4	4	4	2	5	1	5	1	3	5	3	8	2	3
113	CAS-Delhi	2	3	2	2	3	9	4		2	1	4	4	2	2	5	1	7	3	3	4	3	8		3
114	Beijing	2	4	4	2	3	3	3	5		7	4	4	4	2	5	1	7	3	3	5	3	7		3
115	NEW1	2	2	4	2	3	2	3	5	2	2	3	4	2	1	5	1	5	3	2	3	3	7	2	2
116	NEW1	2	2	4	2	3	2	3	5	2	2	3	4	2	1	5	1	6	3	2	3	3	6	2	2
117	CAS-Delhi	2	6	2	2	3	5	4	4	2			4	2	2	5	1	7	3	3	3	3	8		3
118	NEW1	2	2	4	2	3	2	3	5	2	2	3	4	2	1	5	1	5	3	2	3	3	5	2	2
119	NEW1	2	2	4	2	2	2	3	5	2	2	2	4	2	1	5	1	5	3	2	3	3	6	2	2
120	CAS-Delhi	2	3	2	2	2	6	4	4	2	2	4	4	2	2	5	1	7	3	3	4	3	5	2	3
121	CAS-Delhi	2	4	2	2	3	6		4		2	2	4	4	2	5	1	9	3	3	5	3	4	2	3
122	NEW1	2	2	4	2	3	2	2	5	2	2	3	4	2	1	5	1	6	3	2	3	3	7	2	2
123	CAS-Delhi	2	3	2	2	4	6	3	4	2	2	4	4	2	2	5	1	8	3	2	5	3	8	1	3
124	CAS-Delhi	2	3	2	2	3	6	3	4	2	2	4	4	2	2	5	1	5	1	3		3	7	2	3
125	Cameroon	2	2	4	2	4	3	1	2	2	4	2	4	2	2	5	1	5	3	3	3	5	5	2	2
126	Cameroon	2	2	4	2	4	3	1	2	2	4	2	4	2	2	5	1	5	3	3	3	5	5	2	2
127	Cameroon	2	2	4	2	4	3	3	2	2	2	2	4	2	2	5	1	5	3	3	3	3	5	2	2
128	CAS-Delhi	2	3	2	2	3	7	4	4	2			4	4	2	5	1	9		3	3	3	4	3	3
129	Beijing	2	4	4	2	3	3	2	5		7	4	4	4	2	5	1	5	1	3	5	3	8	2	
130	NEW1	2	2	4	2	3	2		3		2	2	4	2	1	5	1	7	3	2	3	3	7	2	2
131	NEW1	2	2	4	2	3	2	3	5	2	1	2	4	2	1	5	1	5	3	2	3	3	7	2	2
132	NEW1	2	2	4	2	2	2		2		2	2	4	2	1	5	1	5	3	2	2	3	10	2	2
133	CAS-Delhi	2	4	2	2	4	6	5	4	2	2	4	4	2	2	5	1	7	3	3	5	3	5	2	3
134	CAS-Delhi	2	3	2	2	3	7	5	6	2	2	3	4	2	2	5	1	5	3	3	4	4	6	5	3
135	Ural1	2	4	4	2	2	6	1	3		2	3	4	4		5	1	1	3		3	3	8		2
136	NEW1	2	2	4	2	3	2	4	5	2	2	3	4	2	1	5	1	5	3	2	3	3	7	2	2
137	Cameroon	2	2	4	2	3	3	4	3	2	4	4	4	2	2	5	0	5	3	3	3	2	6	2	2
138	NEW1	2	2	4	2	2	2	2	3		2	3	4	2	1	5	1	5	3	2	3	3	8		2
139	CAS-Delhi	2	4	2	2	2	3	4	3		2	4	4	4	2	5	1	7	3	3	5	4	3		3
140	CAS-Delhi	2	5	2	3	3	5	4	4			3	4	2	2	5	1	7	3	3	5	3	10	4	2
141	Cameroon	2	2	4	2	3	5	3	3	3	4	4	4	2	2	5	0	5	3	3	3	2	6	2	3
142	CAS-Delhi	2	2	2	2	3	6	4	4	3	2	4	4	2	2	5	1	7	3	3	5	4	9	2	3
143	NEW1	2	2	4	2	3	2	3	5	3	2	3	4	2	1	5	1	5	3	2	3	3	8		2
144	CAS-Delhi	2	7	2	2	3	5	4	4		2		4	2	2	5	1	7	3	3	5	3	9	5	4
145	Haarlem	2	2	4	2	2		2	3		2	4	4	5	2	5	1	3	2	3	3	2	4	4	2
146	CAS-Delhi	2	5	2	2	3	9	4	4	2	1	4	4	2	2	5	1	8	3	3	3	3	8	3	3
147	CAS-Delhi	2	5	2	2	5	5	4	4		2	4	4	2	2	5	1	8	2	2	3	3	8	2	3
148	CAS-Delhi	2	5	2	2	2	6	3	4	2	2	4	4	2	2	5	1	6	2	3	3	3	3		3
149	Uganda	2	1	5	2	2	3	2	3	2	3	4	4	2	2	3	1	5	3	2	3	4	7		2
150	NEW1	2	2	4	2	3	2	3	5	2	2	3	4	2	1	5	1	5	3	2	3	2	7	2	2
151	CAS-Delhi	2	5	2	2	3	5	4	4	2	2	3	4	2	2	5	1	8	3	3	3	3	8	3	3
152	NEW1	2	2	4	2	3	2	3	5	2	2	2	4	2	1	5	1	6	3	2	3	3	7	2	2
153	Beijing	2	2	4	2	3	3	3	5	2	5	4	4	5	2	5	1	5	3	3	3	3	8		3
154	CAS-Delhi	2	4	2	2	3	8	4	4		2	2	4	6	2	5	1	9	3	2	3	3	5		3
155	CAS-Delhi	2	4	2	2	3	7	4	2	2	2	2	4	5	2	5	1	9	3	3	3	3	5		4
156	CAS-Delhi	2		2	2	3	7	4	2		2	2	4	2	2	5	1	9	3	3	3	3	5		4
157	NEW1	2	2	4	2	6	2	3	5	2	3	3	4	2	1	5	1	3	3	3	3	3	6	2	2
158	NEW1	2	2	4	2	6	2	3	5	2	3	3	4	2	1	5	1	3	3	3	3	3	6	2	2
159	NEW1	2	2	4	2	6	2	3	5	2	3	3	4	2	1	5	1	3	3	3	3	3	6	2	2
160	NEW1	2	2	4	2	6	2	3	5	2	3	3	4	2	1	5	1	3	3	3	3	3	6	2	2
161	NEW1	2	2	4	2	6	2	3	5	2	3	3	4	2	1	5	1	3	3	3	3	3	6	2	2
162	NEW1	2	2	4	2	6	2	3	5	2	3	3	4	2	1	5	1	3	3	3	3	3	6	2	2
163	NEW1	2	2	4	2	6	2	3	5	2	3	3	4	2	1	5	1	3	3	3	3	3	6	2	2
164	NEW1	2	2	4	2	6	2	3	5	2	3	3	4	2	1	5	1	3	3	3	3	3	6	2	2

ID	Spoligo 1	Spoligo 2	Spoligo 3	Spoligo 4	Spoligo 5	Spoligo 6	Spoligo 7	Spoligo 8	Spoligo 9	Spoligo 10	Spoligo 11	Spoligo 12	Spoligo 13	Spoligo 14	Spoligo 15	Spoligo 16	Spoligo 17	Spoligo 18	Spoligo 19	Spoligo 20	Spoligo 21	Spoligo 22	Spoligo 23	Spoligo 24	Spoligo 25	Spoligo 26	Spoligo 27	Spoligo 28	Spoligo 29	Spoligo 30	Spoligo 31	Spoligo 32	Spoligo 33	Spoligo 34	Spoligo 35	Spoligo 36	Spoligo 37	Spoligo 38	Spoligo 39	Spoligo 40	Spoligo 41	Spoligo 42	Spoligo 43
1	1	1	1	0	0	0	0	1	1	1	1	1	1	1	1	1	1	1	1	1	1	1	0	0	0	0	0	0	0	0	0	0	0	0	1	1	1	1	1	1	1	1	1
2	1	0	0	1	1	1	1	1	1	1	1	1	1	1	1	1	1	1	1	1	1	1	1	1	1	1	1	1	0	0	0	1	0	0	0	0	1	1	1	1	1	1	1
3	1	1	1	0	0	0	0	1	1	1	1	1	1	1	1	1	1	1	1	1	1	1	0	0	0	0	0	0	0	0	0	0	0	0	1	1	1	1	1	1	1	1	1
4	0	0	0	0	0	0	0	0	0	0	0	0	0	0	0	0	0	0	0	0	0	0	0	0	0	0	0	0	0	0	0	0	0	0	1	1	1	1	1	1	1	1	1
5	1	1	1	1	1	1	1	1	1	1	1	1	1	1	1	1	1	1	1	1	1	1	1	1	1	1	1	1	1	1	1	1	0	0	0	0	1	1	1	1	1	1	1
6	0	0	0	0	0	0	0	0	0	0	0	0	0	0	0	0	0	0	0	0	0	0	0	0	0	0	0	0	0	0	0	0	0	0	1	1	1	1	1	1	1	1	1
7	1	1	1	1	1	1	1	1	1	1	1	1	1	1	1	1	1	1	1	1	1	1	1	1	1	1	1	1	1	1	1	1	0	0	0	0	1	1	1	1	1	1	1
8	1	0	1	1	1	1	1	1	1	1	1	1	1	1	1	1	1	1	1	1	1	1	0	0	0	0	0	0	0	0	0	1	0	0	0	0	1	1	1	1	1	1	1
9	1	1	1	0	0	0	0	1	1	1	1	1	1	1	1	1	1	1	1	1	1	1	0	0	0	0	0	0	0	0	0	0	0	0	1	1	0	0	1	0	0	1	1
10	1	0	1	1	1	1	1	1	1	1	1	1	1	1	1	1	1	1	1	1	1	1	1	1	1	1	1	1	0	0	0	1	0	0	0	0	1	1	1	1	1	1	1
11	1	1	1	0	0	0	0	1	1	1	1	1	1	1	1	1	1	1	1	1	1	1	0	0	0	0	0	0	0	0	0	0	0	0	1	1	1	1	1	1	1	1	1
12	0	0	0	0	0	0	0	0	0	0	0	0	0	0	0	0	0	0	0	0	0	0	0	0	0	0	0	0	0	0	0	0	0	0	1	1	1	1	1	1	1	1	1
13	1	1	1	1	1	1	1	1	1	1	1	1	1	1	1	1	1	1	1	1	1	1	1	1	1	1	1	1	1	1	0	1	0	0	0	0	1	1	1	1	1	1	1
14	1	1	1	0	0	0	0	1	1	1	1	1	1	1	1	1	1	1	1	1	1	1	0	0	0	0	0	0	0	0	0	0	0	0	1	1	1	1	1	1	1	1	1
15	1	1	1	1	1	1	1	1	1	1	1	1	1	1	1	1	1	1	1	1	1	1	1	1	1	1	1	1	1	1	1	1	0	0	0	0	1	1	1	1	1	1	1
16	1	1	1	1	1	1	1	1	1	1	1	1	1	1	1	1	1	1	1	1	1	1	1	1	1	1	1	1	0	0	0	1	0	0	0	0	1	1	1	1	1	1	1
17	1	1	1	1	1	1	1	1	1	1	1	0	1	1	1	1	1	1	1	1	1	1	1	1	1	1	1	1	1	0	0	0	0	0	0	0	1	1	1	1	1	1	1
18	1	0	1	1	1	1	1	1	1	1	1	1	1	1	1	1	1	1	0	1	1	1	1	1	1	1	1	1	0	0	0	1	0	0	0	0	1	1	1	1	1	1	1
19	0	0	0	0	0	0	0	0	0	0	0	0	0	0	0	0	0	0	0	0	0	0	0	0	0	0	0	0	0	0	0	0	0	0	1	1	1	1	1	1	1	1	1
20	0	0	0	0	0	0	0	0	0	0	0	0	0	0	0	0	0	0	0	0	0	0	0	0	0	0	0	0	0	0	0	0	0	0	1	1	1	1	1	1	1	1	1
21	1	0	1	1	1	1	1	1	1	1	1	1	1	1	1	1	1	1	1	1	1	1	1	1	1	1	0	1	0	0	0	1	0	0	0	0	1	1	1	1	1	1	1
22	1	1	1	0	0	0	0	1	1	1	1	0	0	1	1	1	1	1	1	1	1	1	0	0	0	0	0	0	0	0	0	0	0	0	1	1	1	1	1	1	1	1	1
23	0	0	0	0	0	0	0	0	0	0	0	0	0	0	0	0	0	0	0	0	0	0	0	0	0	0	0	0	0	0	0	0	0	0	1	1	1	1	1	1	1	1	1
24	1	1	1	1	1	1	1	1	1	1	1	1	1	1	1	1	1	1	1	1	1	1	1	1	1	1	1	1	1	1	0	1	0	0	0	0	1	1	1	1	1	1	1
25	1	1	1	1	1	1	1	1	1	1	1	1	1	1	1	1	1	1	1	1	1	1	1	1	1	1	1	1	1	1	0	1	0	0	0	0	1	1	1	1	1	1	1
26	1	1	1	0	0	0	0	1	1	1	1	1	1	1	1	1	1	1	1	1	1	1	0	0	0	0	0	0	0	0	0	0	0	0	1	1	0	0	1	1	1	1	1
27	1	1	1	1	1	1	1	1	1	1	1	0	1	1	1	1	1	1	1	1	1	1	1	1	1	1	1	1	1	0	0	0	0	0	0	0	1	1	1	1	1	1	1
28	1	1	1	1	1	1	1	1	1	1	1	1	1	1	1	1	1	1	0	0	0	0	0	0	0	0	0	0	0	0	0	0	0	0	0	0	0	0	0	0	0	1	1
29	1	1	1	1	1	1	1	1	1	1	1	1	1	1	1	1	1	1	1	1	1	1	1	1	1	1	1	1	0	0	0	1	0	0	0	0	1	1	1	1	1	1	1
30	1	1	1	1	1	1	1	1	1	1	1	1	1	1	1	1	1	1	1	1	1	1	1	1	1	1	1	1	1	1	1	1	0	0	0	0	1	0	1	1	1	1	1
31	1	1	1	0	0	0	0	1	1	1	1	1	1	1	1	1	1	1	0	0	1	1	0	0	0	0	0	0	0	0	0	0	0	0	1	1	1	1	1	1	1	1	1
32	1	0	1	1	1	1	1	1	1	1	1	1	1	1	1	1	1	1	1	1	1	1	1	1	1	1	1	1	0	0	0	1	0	0	0	0	1	1	1	1	1	1	1
33	1	1	1	1	1	1	1	1	1	1	1	1	1	1	1	1	1	1	1	1	1	1	0	0	0	1	1	1	1	1	1	1	0	0	0	0	1	1	1	1	1	1	1
34	1	0	1	1	1	1	1	1	1	1	1	1	1	1	1	1	1	1	1	1	1	1	1	1	1	1	1	1	0	0	0	1	0	0	0	0	1	1	1	1	1	1	1
35	1	0	0	1	1	1	1	1	1	1	1	1	1	1	1	1	1	1	1	1	1	1	1	1	1	1	1	1	0	0	0	1	0	0	0	0	1	1	1	1	1	1	1
36	1	0	1	1	1	1	1	1	1	1	1	1	1	1	1	1	1	1	1	1	1	1	1	1	1	1	1	1	0	0	0	1	0	0	0	0	1	1	1	1	1	1	1
37	1	1	1	1	1	1	1	1	1	1	1	1	1	1	1	1	1	1	1	1	1	1	1	1	1	1	1	1	0	0	0	1	0	0	0	0	1	1	1	1	1	1	1
38	0	0	0	0	0	0	0	0	0	0	0	0	0	0	0	0	0	0	0	0	0	0	0	0	0	0	0	0	0	0	0	0	0	0	1	1	1	1	1	1	1	1	1
39	1	1	1	0	0	0	0	1	1	1	1	1	1	1	1	1	1	1	1	1	1	1	0	0	0	0	0	0	0	0	0	0	0	0	1	1	1	1	1	1	1	1	1
40	1	0	1	1	1	1	1	1	1	1	1	1	1	1	1	1	1	1	1	1	1	1	1	1	1	1	1	1	0	0	0	1	0	0	0	0	1	1	1	1	1	1	1
41	1	0	1	1	1	1	1	1	1	1	1	1	1	1	1	1	1	1	1	1	1	1	1	1	1	1	0	1	0	0	0	1	0	0	0	0	1	1	1	1	1	1	1
42	0	0	0	0	0	0	0	0	0	0	0	0	0	0	0	0	0	0	0	0	0	0	0	0	0	0	0	0	0	0	0	0	0	0	1	1	1	1	1	1	1	1	1
43	1	0	1	1	1	1	1	1	1	1	1	1	1	1	1	1	1	1	1	1	1	1	1	1	1	1	1	1	0	0	0	1	0	0	0	0	1	1	1	1	1	1	1
44	1	1	1	0	0	0	0	1	1	1	1	1	1	1	1	1	1	1	1	1	1	1	0	0	0	0	0	0	0	0	0	0	0	0	1	1	1	1	1	1	1	1	1
45	1	1	1	0	0	0	0	1	1	1	1	1	1	1	1	1	1	1	1	1	1	1	0	0	0	0	0	0	0	0	0	0	0	0	1	1	1	1	1	1	1	1	1
46	1	1	1	0	0	0	0	1	1	1	1	1	1	1	1	1	1	1	1	1	1	1	0	0	0	0	0	0	0	0	0	0	0	0	1	1	1	1	1	1	1	1	1
47	1	1	1	0	0	0	0	1	1	1	1	1	1	1	1	1	1	1	1	1	1	1	0	0	0	0	0	0	0	0	0	0	0	0	1	1	1	1	1	1	1	1	1
48	0	0	0	0	0	0	0	0	0	0	0	0	0	0	0	0	0	0	0	0	0	0	0	0	0	0	0	0	0	0	0	0	0	0	1	1	1	1	1	1	1	1	1
49	1	0	1	1	1	1	1	1	1	1	1	1	1	1	1	1	1	1	0	1	1	1	1	1	1	1	1	1	0	0	0	1	0	0	0	0	1	1	1	1	1	1	1
50	1	1	1	0	0	0	0	1	1	1	1	0	1	1	1	1	1	1	1	1	1	1	0	0	0	0	0	0	0	0	0	0	0	0	1	1	1	1	1	1	1	1	1
51	0	0	0	0	0	0	0	0	0	0	0	0	0	0	0	0	0	0	0	0	0	0	0	0	0	0	0	0	0	0	0	0	0	0	1	1	1	1	1	1	1	1	1
52	0	0	0	0	0	0	0	0	0	0	0	0	0	0	0	0	0	0	0	0	0	0	0	0	0	0	0	0	0	0	0	0	0	0	1	1	1	1	1	1	1	1	1
53	1	1	1	0	0	0	0	1	1	1	1	1	1	1	1	1	1	1	1	1	1	1	0	0	0	0	0	0	0	0	0	0	0	0	1	1	1	1	1	1	1	1	1
54	1	1	1	1	1	1	1	1	1	1	1	0	1	1	1	1	1	1	1	1	1	1	1	1	1	1	1	1	1	0	0	0	0	0	0	0	1	1	1	1	1	1	1
55	1	1	1	1	1	1	1	1	1	1	1	1	1	1	1	1	1	1	1	1	1	1	0	0	0	1	1	1	1	1	1	1	0	0	0	0	1	1	1	1	1	1	1
56	1	1	1	0	0	0	0	1	1	1	1	1	1	1	1	1	1	1	1	1	1	1	0	0	0	0	0	0	0	0	0	0	0	0	1	1	1	1	1	1	1	1	1
57	1	1	1	1	1	1	1	1	1	1	1	1	1	1	1	1	1	1	1	1	1	1	1	1	1	1	1	1	1	1	1	0	0	0	0	0	1	1	1	1	1	1	1
58	1	0	1	1	1	1	1	1	1	1	1	1	1	1	1	1	1	1	1	1	1	1	1	1	1	1	1	1	0	0	0	1	0	0	0	0	1	1	1	1	1	1	1
59	1	0	1	1	1	1	1	1	1	1	1	1	1	1	1	1	1	1	0	1	1	1	1	1	1	1	1	1	0	0	0	1	0	0	0	0	1	1	1	1	1	1	1
60	1	0	1	1	1	1	1	1	1	1	1	1	1	1	1	1	1	1	1	1	1	1	1	1	1	1	1	1	0	0	0	1	0	0	0	0	1	1	1	1	1	1	1
61	1	1	1	0	0	0	0	1	1	1	1	1	1	1	1	1	1	1	1	1	1	1	0	0	0	0	0	0	0	0	0	0	0	0	1	1	1	1	1	1	1	1	1
62	1	1	1	1	1	1	1	1	0	0	1	1	1	1	1	1	1	1	1	1	1	1	1	1	1	1	1	1	0	0	0	1	0	0	0	0	1	1	1	1	1	1	1
63	1	1	1	0	0	0	0	1	1	1	1	1	1	1	1	1	1	1	1	1	1	1	0	0	0	0	0	0	0	0	0	0	0	0	1	1	0	0	1	0	0	1	1
64	1	1	1	0	0	0	0	1	1	1	1	1	1	1	1	1	1	1	1	1	1	1	0	0	0	0	0	0	0	0	0	0	0	0	1	1	1	1	1	1	1	1	1
65	1	1	1	0	0	0	0	1	1	1	1	1	1	1	1	1	1	1	1	1	1	1	0	0	0	0	0	0	0	0	0	0	0	0	1	1	1	1	1	1	1	1	1
66	1	1	1	0	0	0	0	1	1	1	1	1	1	1	1	1	1	1	1	1	1	1	0	0	0	0	0	0	0	0	0	0	0	0	0	0	0	0	0	0	0	0	0
67	1	0	1	1	1	1	1	1	1	1	1	1	1	1	1	1	1	1	1	1	1	1	1	1	1	1	1	1	0	0	0	1	0	0	0	0	1	1	1	1	1	1	1
68	1	0	1	1	1	1	1	1	1	1	1	1	1	1	1	1	1	1	1	1	1	1	1	1	1	1	1	1	0	0	0	1	0	0	0	0	1	1	1	0	1	1	1
69	1	1	1	1	1	1	1	1	1	1	1	0	1	1	1	1	1	1	1	1	1	1	1	1	1	1	1	1	1	0	0	0	0	0	0	0	1	1	1	1	1	1	1
70	0	0	0	0	0	0	0	0	0	0	0	0	0	0	0	0	0	0	0	0	0	0	0	0	0	0	0	0	0	0	0	0	0	0	1	1	1	1	1	1	1	1	1
71	1	0	1	1	1	1	1	1	1	1	1	1	1	1	1	1	1	1	1	1	1	1	1	1	1	1	1	1	0	0	0	1	0	0	0	0	1	1	1	1	1	1	1
72	1	0	1	1	1	1	1	1	1	1	1	1	1	1	1	1	1	1	1	1	1	1	1	1	1	1	1	1	0	0	0	1	0	0	0	0	1	1	1	1	1	1	1
73	1	1	1	0	0	0	0	1	1	1	1	1	1	1	1	1	1	1	1	1	1	1	0	0	0	0	0	0	0	0	0	0	0	0	1	1	0	0	1	1	1	1	1
74	1	1	1	1	1	1	1	1	1	1	1	1	1	1	1	1	1	1	1	1	1	1	1	1	1	1	1	1	1	1	1	0	0	0	0	0	1	0	1	1	1	1	1
75	1	1	1	1	1	1	1	1	1	1	1	0	1	1	1	1	1	1	1	1	1	1	0	0	0	1	1	1	1	1	1	1	0	0	0	0	1	1	1	1	1	1	1
76	1	0	1	1	1	1	1	1	1	1	1	1	1	1	1	1	1	1	1	1	1	1	1	1	1	1	1	1	0	0	0	1	0	0	0	0	1	1	1	1	1	1	1
77	1	1	1	1	1	1	1	1	1	1	1	1	1	1	1	1	1	1	1	1	1	1	1	1	1	1	1	1	1	1	1	0	0	0	0	0	1	1	1	1	1	1	1
78	1	0	1	1	1	1	1	1	1	1	1	1	1	1	1	1	1	1	1	1	1	1	1	1	1	1	1	1	0	0	0	1	0	0	0	0	1	1	1	1	1	1	1
79	1	0	1	1	1	1	1	1	1	1	1	1	1	1	1	1	1	1	1	1	1	1	1	1	1	1	1	1	0	0	0	1	0	0	0	0	1	1	1	1	1	1	1
80	1	1	1	0	0	0	0	1	1	1	1	1	1	1	1	1	1	1	1	1	1	1	0	0	0	0	0	0	0	0	0	0	0	0	1	1	1	1	1	1	1	1	1
81	1	0	1	1	1	1	1	1	1	1	1	1	1	1	1	1	1	1	1	1	1	1	1	1	1	1	0	1	0	0	0	1	0	0	0	0	1	1	1	1	1	1	1
82	1	1	1	0	0	0	0	1	1	1	1	1	1	1	1	1	1	1	1	1	1	1	0	0	0	0	0	0	0	0	0	0	0	0	1	1	0	0	1	1	1	1	1
83	1	0	1	1	1	1	1	1	1	1	1	1	1	1	1	1	1	1	1	1	1	1	1	1	1	1	1	1	0	0	0	1	0	0	0	0	1	1	1	1	1	1	1
84	1	1	1	0	0	0	0	1	1	1	1	1	1	1	1	1	1	1	1	1	1	1	0	0	0	0	0	0	0	0	0	0	0	0	1	1	0	0	0	1	1	1	1
85	1	1	0	1	1	1	1	1	1	1	1	1	1	1	1	1	1	1	1	1	1	1	1	1	1	1	1	1	0	0	0	1	0	0	0	0	1	1	1	1	1	1	1
86	1	1	1	1	1	1	1	1	1	1	1	1	1	0	0	0	0	0	0	0	0	0	0	0	0	1	0	0	0	0	0	0	0	0	0	0	1	1	1	1	1	1	1
87	0	0	0	0	0	0	0	0	0	0	0	0	0	0	0	0	0	0	0	0	0	0	0	0	0	0	0	0	0	0	0	0	0	0	1	1	1	1	1	1	1	1	1
88	1	0	1	1	1	1	1	1	1	1	1	1	1	1	1	1	1	1	1	1	1	1	1	1	1	1	1	1	0	0	0	1	0	0	0	0	1	1	1	0	0	0	0
89	1	0	1	1	1	1	1	1	1	1	1	1	1	1	1	1	1	0	1	1	1	1	1	1	1	1	1	1	0	0	0	1	0	0	0	0	1	1	1	1	1	1	1
90	1	1	1	1	1	1	1	1	1	1	1	1	1	1	1	1	1	1	1	1	1	1	0	0	0	1	1	1	1	1	1	1	0	0	0	0	1	1	1	1	1	1	1
91	1	1	1	0	0	0	0	1	1	1	1	1	1	0	0	0	0	0	0	0	0	0	0	0	0	0	0	0	0	0	0	0	0	0	1	1	1	1	1	1	1	0	0
92	1	0	1	1	1	1	1	1	1	1	1	1	1	1	1	1	1	1	0	1	1	1	1	1	1	1	0	0	0	0	0	0	0	0	0	0	0	0	0	0	0	1	1
93	1	1	1	1	1	1	1	1	1	1	1	1	1	1	1	1	1	1	1	1	1	1	0	0	0	1	1	1	1	1	1	1	0	0	0	0	1	1	1	1	1	1	1
94	1	1	1	1	1	1	1	1	0	0	1	1	1	1	1	1	1	1	1	1	1	1	1	1	1	1	1	1	0	0	0	1	0	0	0	0	1	1	1	1	1	1	1
95	1	1	1	0	0	0	0	1	1	1	1	1	1	1	1	1	1	1	1	1	1	1	0	0	0	0	0	0	0	0	0	0	0	0	1	1	1	1	1	1	1	1	1
96	0	0	0	0	0	0	0	0	0	0	0	0	0	0	0	0	0	0	0	0	0	0	0	0	0	0	0	0	0	0	0	0	0	0	1	1	1	1	1	1	1	1	1
97	1	0	1	1	1	1	1	1	1	1	1	1	1	1	1	1	1	1	0	1	1	1	1	1	1	1	1	1	0	0	0	1	0	0	0	0	1	1	1	1	1	1	1
98	1	1	1	0	0	0	0	1	1	1	1	1	1	1	1	1	1	1	1	1	1	1	0	0	1	1	1	1	1	1	1	1	0	0	0	0	1	1	1	1	1	1	1
99	1	1	1	0	0	0	0	1	1	1	1	1	1	1	1	1	1	1	1	1	1	1	0	0	0	0	0	0	0	0	0	0	0	0	1	1	1	1	1	1	1	1	1
100	1	0	1	1	1	1	1	1	1	1	1	1	1	1	1	1	1	1	1	1	1	1	1	1	1	1	1	1	0	0	0	1	0	0	0	0	1	1	1	1	1	1	1
101	1	1	1	0	0	0	0	1	1	1	1	1	1	1	1	1	1	1	1	1	1	1	0	0	0	0	0	0	0	0	0	0	0	0	1	1	1	1	1	1	1	1	0
102	1	1	1	0	0	0	0	1	1	1	1	1	1	1	1	1	1	1	1	1	1	1	0	0	0	0	0	0	0	0	0	0	0	0	1	1	1	1	1	1	1	1	1
103	1	0	1	1	1	1	1	1	1	1	1	1	1	1	1	1	1	1	1	1	1	1	1	1	1	1	1	1	0	0	0	1	0	0	0	0	1	1	1	1	1	1	1
104	1	1	1	0	0	0	0	1	1	1	1	1	1	1	1	1	1	1	1	1	1	1	0	0	0	0	0	0	0	0	0	0	0	0	0	1	1	1	1	1	0	1	1
105	0	0	0	0	0	0	0	0	0	0	0	0	0	0	0	0	0	0	0	0	0	0	0	0	0	0	0	0	0	0	0	0	0	0	1	1	1	1	1	1	1	1	1
106	1	0	1	1	1	1	1	1	1	1	1	1	1	1	1	1	1	1	1	1	1	1	1	1	1	1	1	1	0	0	0	1	0	0	0	0	1	1	1	1	1	1	1
107	1	0	1	1	1	1	1	1	1	1	1	1	1	1	1	1	1	1	1	1	1	1	1	1	1	1	1	1	0	0	0	1	0	0	0	0	1	1	1	1	1	1	1
108	1	0	1	1	1	1	1	1	1	1	1	1	1	1	1	1	1	1	1	1	1	1	1	1	1	1	1	1	0	0	0	1	0	0	0	0	1	1	1	1	1	1	1
109	1	1	1	0	0	0	0	1	1	1	1	1	1	1	1	1	1	1	1	1	1	1	0	0	0	0	0	0	0	0	0	0	0	0	1	1	0	0	1	1	1	1	1
110	1	1	1	0	0	0	0	1	1	1	1	1	1	1	1	1	1	1	1	1	1	1	0	0	0	0	0	0	0	0	0	0	0	0	1	1	1	1	1	1	1	1	1
111	1	0	1	1	1	1	1	1	1	1	1	1	1	1	1	1	1	1	1	1	1	1	1	1	1	1	1	1	0	0	0	1	0	0	0	0	1	1	1	1	1	1	1
112	0	0	0	0	0	0	0	0	0	0	0	0	0	0	0	0	0	0	0	0	0	0	0	0	0	0	0	0	0	0	0	0	0	0	1	1	1	1	1	1	1	1	1
113	1	1	1	0	0	0	0	1	1	1	1	1	1	1	1	1	1	1	1	0	0	0	0	0	0	0	0	0	0	0	0	0	0	0	1	1	1	1	1	1	1	1	1
114	0	0	0	0	0	0	0	0	0	0	0	0	0	0	0	0	0	0	0	0	0	0	0	0	0	0	0	0	0	0	0	0	0	0	1	1	1	1	1	1	1	1	1
115	1	0	1	1	1	1	1	1	1	1	1	1	1	1	1	1	1	1	1	1	1	1	1	1	1	1	1	1	0	0	0	1	0	0	0	0	1	1	1	1	1	1	1
116	1	0	1	1	1	1	1	1	1	1	1	1	1	1	1	1	1	1	1	1	1	1	1	1	1	1	1	1	0	0	0	1	0	0	0	0	1	1	1	1	1	1	1
117	1	1	1	0	0	0	0	1	1	1	1	1	1	1	1	1	1	1	1	1	1	1	0	0	0	0	0	0	0	0	0	0	0	0	1	1	1	1	1	1	1	1	1
118	1	0	1	1	1	0	0	1	1	1	1	1	1	1	1	1	1	1	1	1	1	1	1	1	1	1	1	1	0	0	0	1	0	0	0	0	1	1	1	1	1	1	1
119	1	0	1	1	1	1	1	1	1	1	1	1	1	1	1	1	1	1	0	1	1	1	1	1	1	1	1	1	0	0	0	1	0	0	0	0	1	1	1	1	1	1	1
120	1	1	1	0	0	0	0	1	1	1	1	1	1	1	1	1	1	1	1	0	0	0	0	0	0	0	0	0	0	0	0	0	0	0	0	1	1	1	1	1	1	1	1
121	1	1	1	0	0	0	0	1	1	1	1	1	1	1	1	1	1	1	1	1	1	1	0	0	0	0	0	0	0	0	0	0	0	0	1	1	1	1	1	1	1	1	1
122	1	0	1	1	1	1	1	1	1	1	1	1	1	1	1	1	1	1	1	1	1	1	1	1	1	1	1	1	0	0	0	1	0	0	0	0	1	1	1	1	1	1	1
123	1	1	1	0	0	0	0	1	1	1	1	1	1	1	1	1	1	1	1	1	1	1	0	0	0	0	0	0	0	0	0	0	0	0	1	1	1	1	1	1	1	1	0
124	1	1	1	0	0	0	0	1	1	1	1	1	1	1	1	1	1	1	1	1	1	1	0	0	0	0	0	0	0	0	0	0	0	0	1	1	1	1	1	1	1	1	1
125	1	1	1	1	1	1	1	1	1	1	1	1	1	1	1	1	1	1	1	1	1	1	0	0	0	1	1	1	1	1	1	1	0	0	0	0	1	1	1	1	1	1	1
126	1	1	1	1	1	1	1	1	1	1	1	1	1	1	1	1	1	1	1	1	1	1	0	0	0	1	1	1	1	1	1	1	0	0	0	0	1	1	1	1	1	1	1
127	1	1	1	1	1	1	1	1	1	1	1	1	1	1	1	1	1	1	1	1	1	1	0	0	0	1	1	1	1	1	1	1	0	0	0	0	1	1	1	1	1	1	1
128	1	1	1	0	0	0	0	1	0	1	1	0	1	1	1	1	1	1	1	1	1	1	0	0	0	0	0	0	0	0	0	0	0	0	1	1	1	1	1	1	1	1	1
129	0	0	0	0	0	0	0	0	0	0	0	0	0	0	0	0	0	0	0	0	0	0	0	0	0	0	0	0	0	0	0	0	0	0	1	1	1	1	1	1	1	1	1
130	1	0	1	1	1	1	1	1	1	1	1	1	1	1	1	1	1	1	1	1	1	1	1	1	1	1	1	1	0	0	0	1	0	0	0	0	1	1	1	1	1	1	1
131	1	0	1	1	1	1	1	1	1	1	1	1	1	1	1	1	1	1	1	1	1	1	1	1	1	1	1	1	0	0	0	1	0	0	0	0	1	1	1	1	1	1	1
132	1	0	1	1	1	1	1	1	1	1	1	1	1	1	1	1	1	1	1	1	1	1	1	1	1	1	1	1	0	0	0	1	0	0	0	0	1	1	1	1	1	1	1
133	1	1	1	0	0	0	0	1	1	1	1	1	1	1	1	1	1	1	1	1	1	1	0	0	0	0	0	0	0	0	0	0	0	0	1	1	1	1	1	1	1	1	1
134	1	1	1	0	0	0	0	1	1	1	1	1	1	1	1	1	1	1	1	1	1	1	0	0	0	0	0	0	0	0	0	0	0	0	1	1	0	0	1	1	1	1	1
135	1	1	1	1	1	1	1	1	1	1	1	1	1	1	1	1	1	1	1	1	1	1	1	1	1	1	1	1	1	1	1	1	0	0	0	0	1	1	1	1	1	1	1
136	1	0	1	1	1	1	1	1	1	1	1	1	1	1	1	1	1	1	1	1	1	1	1	1	1	1	1	1	0	0	0	1	0	0	0	0	1	1	1	1	1	1	1
137	1	1	1	1	1	1	1	1	1	1	1	1	1	1	1	1	1	1	1	1	1	1	0	0	0	1	1	1	1	1	1	1	0	0	0	0	1	1	1	0	1	1	1
138	1	0	1	1	1	1	1	1	1	1	1	1	1	1	1	1	1	1	0	1	1	1	1	1	1	1	1	1	0	0	0	1	0	0	0	0	1	1	1	1	1	1	1
139	1	1	1	0	0	0	0	0	1	1	1	1	1	1	1	1	1	1	1	1	1	1	0	0	1	1	1	1	1	1	1	0	0	0	0	0	1	1	1	1	1	1	1
140	1	1	1	0	0	0	0	1	1	1	1	1	1	1	1	1	1	1	1	1	1	1	0	0	0	0	0	0	0	0	0	0	0	0	1	1	1	1	1	1	1	1	1
141	1	1	1	1	1	1	1	1	1	1	1	1	1	1	1	1	1	1	1	1	1	1	0	0	0	1	1	1	1	1	1	1	0	0	0	0	1	1	1	1	1	1	1
142	1	1	1	0	0	0	0	1	1	1	1	1	1	0	0	0	0	1	0	0	0	0	0	0	0	0	0	0	0	0	0	0	0	0	1	1	1	1	1	1	1	0	0
143	1	0	1	1	1	1	1	1	1	1	1	1	1	1	1	1	1	1	1	1	1	1	1	1	1	1	0	0	0	0	0	0	0	0	0	0	0	0	0	0	0	1	1
144	1	1	1	0	0	0	0	1	1	1	1	1	1	1	1	1	1	1	1	1	1	1	0	0	0	0	0	0	0	0	0	0	0	0	1	1	1	1	1	1	1	1	1
145	1	1	1	1	1	1	1	1	0	0	1	1	1	1	1	1	1	1	1	1	1	1	1	1	1	1	1	1	0	0	0	1	0	0	0	0	1	1	1	1	1	1	1
146	1	1	1	0	0	0	0	1	1	1	1	1	1	1	1	1	1	1	1	1	1	1	0	0	0	0	0	0	0	0	0	0	0	0	1	1	1	1	1	1	1	1	1
147	1	1	1	0	0	0	0	1	1	1	1	1	1	1	1	1	1	1	1	1	1	1	0	0	0	0	0	0	0	0	0	0	0	0	1	1	1	1	1	1	1	1	1
148	1	1	1	0	0	0	0	1	1	1	1	1	1	1	1	1	1	1	1	1	1	1	0	0	0	0	0	0	0	0	0	0	0	0	1	1	1	1	1	1	1	1	1
149	1	1	1	1	1	1	1	1	1	1	1	1	1	1	1	1	1	1	1	1	1	1	1	1	1	1	1	1	1	1	1	0	0	0	0	0	1	0	1	1	1	1	1
150	1	0	1	1	1	0	1	1	1	1	1	1	1	1	1	1	1	1	1	1	1	1	1	1	1	1	1	1	0	0	0	1	0	0	0	0	1	1	1	1	1	1	1
151	1	1	1	0	0	0	0	0	1	1	1	1	1	1	1	1	1	1	1	1	1	1	0	0	1	1	1	1	1	1	1	0	0	0	0	0	1	1	1	1	1	1	1
152	1	0	1	1	1	0	1	1	1	1	1	1	1	1	1	1	1	1	1	1	1	1	1	1	1	1	1	1	0	0	0	1	0	0	0	0	1	1	1	1	1	1	1
153	0	0	0	0	0	0	0	0	0	0	0	0	0	0	0	0	0	0	0	0	0	0	0	0	0	0	0	0	0	0	0	0	0	0	1	1	1	1	1	1	1	1	1
154	1	1	1	0	0	0	0	1	1	1	1	1	1	1	1	1	1	1	1	1	1	1	0	0	0	0	0	0	0	0	0	0	0	0	1	1	1	1	1	1	1	1	1
155	1	1	1	0	0	0	0	1	1	1	1	1	1	1	1	1	1	1	1	1	1	1	0	0	0	0	0	0	0	0	0	0	0	0	1	1	1	1	1	1	1	1	1
156	1	1	1	0	0	0	0	1	1	1	1	1	1	1	1	1	1	1	1	1	1	1	0	0	0	0	0	0	0	0	0	0	0	0	1	1	1	1	1	1	1	1	1
157	1	0	1	1	1	0	1	1	1	1	1	1	1	1	1	1	1	1	1	1	1	1	1	1	1	1	1	1	0	0	0	1	0	0	0	0	1	1	1	1	1	1	1
158	1	0	1	1	1	0	1	1	1	1	1	1	1	1	1	1	1	1	1	1	1	1	1	1	1	1	1	1	0	0	0	1	0	0	0	0	1	1	1	1	1	1	1
159	1	0	1	1	1	0	1	1	1	1	1	1	1	1	1	1	1	1	1	1	1	1	1	1	1	1	1	1	0	0	0	1	0	0	0	0	1	1	1	1	1	1	1
160	1	0	1	1	1	0	1	1	1	1	1	1	1	1	1	1	1	1	1	1	1	1	1	1	1	1	1	1	0	0	0	1	0	0	0	0	1	1	1	1	1	1	1
161	1	0	1	1	1	0	1	1	1	1	1	1	1	1	1	1	1	1	1	1	1	1	1	1	1	1	1	1	0	0	0	1	0	0	0	0	1	1	1	1	1	1	1
162	1	0	1	1	1	0	1	1	1	1	1	1	1	1	1	1	1	1	1	1	1	1	1	1	1	1	1	1	0	0	0	1	0	0	0	0	1	1	1	1	1	1	1
163	1	0	1	1	1	0	1	1	1	1	1	1	1	1	1	1	1	1	1	1	1	1	1	1	1	1	1	1	0	0	0	1	0	0	0	0	1	1	1	1	1	1	1
164	1	0	1	1	1	0	1	1	1	1	1	1	1	1	1	1	1	1	1	1	1	1	1	1	1	1	1	1	0	0	0	1	0	0	0	0	1	1	1	1	1	1	1

165	NEW1	2	2	4	2	6	2	3	5	2	3	3	4	2	1	5	1	3	3	3	3	3	6	2	2	1	0	1	1	1	0	1	1	1	1	1	1	1	1	1	1	1	1	1	1	1	1	1	1	1	1	1	1	0	0	0	1	0	0	0	0	1	1	1	1	1	1	1
166	Haarlem	2					4				3	3	4	2	2	6	1		3	3				2	2	1	1	1	1	1	1	1	1	1	1	1	0	1	1	1	1	1	1	1	1	1	1	1	1	1	1	1	1	1	0	0	0	0	0	0	0	1	1	1	1	1	1	1
167	LAM	2		3	3	1	3	5	3	1	3	1	4	2	2	6	1	6	3	6	3	4	8	2	2	1	1	1	1	1	1	1	1	1	1	1	1	1	1	1	1	1	1	1	1	0	0	0	0	1	1	1	1	1	1	1	1	0	0	0	0	1	1	1	1	1	1	1
168	CAS-Delhi	2	3	2	2		6	5	5	2			4	2	2	5	1	5	3	3	6	3	6	2	2	1	1	1	0	0	0	0	0	1	1	1	1	1	1	1	1	1	1	1	1	1	1	0	0	1	1	1	1	1	1	1	1	1	0	0	0	0	1	1	1	1	1	1
169	CAS-Delhi	2	3	2	2		6	5	5	2			4	2	2	5	1	5	3	3	6	3	6	2	2	1	1	1	0	0	0	0	0	1	1	1	1	1	1	1	1	1	1	1	1	1	1	0	0	1	1	1	1	1	1	1	1	1	0	0	0	0	1	1	1	1	1	1
170	Uganda	2			2				2	2	1	4	4	2	2	5	1	5	3	3		3	2	2	2	1	1	1	1	1	1	1	1	1	1	1	1	1	1	1	1	1	1	1	1	1	1	1	1	1	1	1	1	1	1	1	1	0	0	0	0	1	0	1	1	1	1	1
171	NEW1	2	3	4	2	3	2	5	5	2	2	3	4	2	1	5	1	5	3	2	3	3	7	2	2	1	0	1	1	1	0	1	1	1	1	1	1	1	1	1	1	1	1	1	1	1	1	1	1	1	1	1	1	0	0	0	1	0	0	0	0	1	1	1	1	1	1	1
172	NEW1	2	3	4	2	3	2	5	5	2	2	3	4	2	1	5	1	5	3	2	3	3	7	2	2	1	0	1	1	1	0	1	1	1	1	1	1	1	1	1	1	1	1	1	1	1	1	1	1	1	1	1	1	0	0	0	1	0	0	0	0	1	1	1	1	1	1	1
173	NEW1	2	2	4	2	3	2	3	4		2	4	4	2	1	1	1	5	3	2	3	3	7	2	2	1	0	1	1	1	0	1	1	1	1	1	1	1	1	1	1	1	1	1	1	1	1	1	1	1	1	1	1	0	0	0	1	0	0	0	0	1	1	1	1	1	1	1
174	NEW1	2	2	4	2	3	2	3	4		2	4	4	2	1	5	1	6	3	2	3	3	7	2	2	1	0	1	1	1	0	1	1	1	1	1	1	1	1	1	1	1	1	1	1	1	1	1	1	1	1	1	1	0	0	0	1	0	0	0	0	1	1	1	1	1	1	1
175	NEW1	2	2	4	2	3	2	3	4		2	4	4	2	1	5	1	6	3	2	3	3	7	2	2	1	0	0	1	1	1	1	1	1	1	1	1	1	1	1	1	1	1	1	1	1	1	1	1	1	1	1	1	0	0	0	1	0	0	0	0	1	1	1	1	1	1	1
176	NEW1	2	2	4	2	3	2	3	4		2	4	4	2	1	5	1	6	3	2	3	3	7	2	2	1	0	0	1	1	1	1	1	1	1	1	1	1	1	1	1	1	1	1	1	1	1	1	1	1	1	1	1	0	0	0	1	0	0	0	0	1	1	1	1	1	1	1
177	CAS-Delhi	2	4	2	2	3	7	4	5	2	2	2	4	4	2	5	1	10	3	2	6	4	4	2	2	1	1	1	0	0	0	0	1	1	1	1	1	1	1	1	1	1	1	1	1	1	1	0	0	0	0	0	0	0	0	0	0	0	0	0	0	1	1	1	1	1	1	1
178	CAS-Delhi	2	3	2	2	3	7	4	5		2	2	4	4	2	5	1	10	3	3	6	3	4	2	2	1	1	1	0	0	0	0	1	1	1	1	1	1	1	1	1	1	1	1	1	1	1	0	0	0	0	0	0	0	0	0	0	0	0	0	0	1	1	1	1	1	1	1
179	CAS-Delhi	2	4	2	2	3	7	4	5		2	2	4	4	2	5	1	10	3	3	6	3	4	2	2	1	1	1	0	0	0	0	1	1	1	1	1	1	1	1	1	1	1	1	1	1	1	0	0	0	0	0	0	0	0	0	0	0	0	0	0	1	1	1	1	1	1	1
180	CAS-Delhi	2	4	2	2	3	7	4	5		2	2	4	4	2	5	1	10	3	3	6	3	4	2	2	1	1	1	0	0	0	0	0	1	1	1	1	1	1	1	1	1	1	1	1	1	1	0	0	1	1	1	1	1	1	1	1	1	0	0	0	0	1	1	1	1	1	1
181	CAS-Delhi	2	4	2	2	3	7	4	5		2	2	4	4	2	5	1	10	3	3	6	3	4	2	2	1	1	1	0	0	0	0	0	1	1	1	1	1	1	1	1	1	1	1	1	1	1	0	0	1	1	1	1	1	1	1	1	1	0	0	0	0	1	1	1	1	1	1
182	CAS-Delhi	2		2	2	3	7	4	5		2	2	4	4	2	5	1	10	3	3	6	3	4	2	2	1	1	1	0	0	0	0	1	1	1	1	1	1	1	1	1	1	1	1	1	1	1	0	0	0	0	0	0	0	0	0	0	0	0	0	0	1	1	1	1	1	1	1
183	CAS-Delhi	2		2	2	3	7	4	5		2	2	4	4	2	5	1	10	3	3	6	3	4	2	2	1	1	1	0	0	0	0	1	1	1	1	1	1	1	1	1	1	1	1	1	1	1	0	0	0	0	0	0	0	0	0	0	0	0	0	0	1	1	1	1	1	1	1
184	CAS-Delhi	2	4	2	2	3	7	4	5	2	2	2	4	4	2	5	1	10	3	3	6	3	4	2	2	1	1	1	0	0	0	0	1	1	1	1	1	1	1	1	1	1	1	1	1	1	1	0	0	0	0	0	0	0	0	0	0	0	0	0	0	1	1	1	1	1	1	1
185	CAS-Delhi	2	4	2	2	3	7	4	5		2	2	4	4	2	5	1	10	3	3	6	3	4	2	2	1	1	1	0	0	0	0	1	1	1	1	1	1	1	1	1	1	1	1	1	1	1	0	0	0	0	0	0	0	0	0	0	0	0	0	0	1	1	1	1	1	1	1
186	CAS-Delhi	2	4	2	2	3	7	4	5		2	2	4	4	2	5	1	10	3	3	6	3	4	2	2	1	1	1	0	0	0	0	1	1	1	1	1	1	1	1	1	1	1	1	1	1	1	0	0	0	0	0	0	0	0	0	0	0	0	0	0	1	1	0	0	1	1	1
187	CAS-Delhi	2	1	2	2	3	7	5	5	2	2	4	4	4	2	6	1	5	3	3	6	3	8	2	2	1	1	1	0	0	0	0	0	1	1	1	1	1	1	1	1	1	1	1	1	1	1	0	0	1	1	1	1	1	1	1	1	1	0	0	0	0	1	1	1	1	1	1
188	CAS-Delhi	2	2	2	2	3	7	4	5		2	2	4	4	2	5	1	5	3	3	6	3	4	2	2	1	1	1	0	0	0	0	0	1	1	1	1	1	1	1	1	1	1	1	1	1	1	0	0	1	1	1	1	1	1	1	1	1	0	0	0	0	1	1	1	1	1	1
189	Uganda	2	2	3	2	3	3	3	2	1	2	2	4	2	1	5	1	5	3	2	6	3	7	2	2	1	1	1	1	1	1	1	1	1	1	1	1	1	1	1	1	1	1	1	1	1	1	1	1	1	1	1	1	1	1	1	1	0	0	0	0	1	1	1	1	1	1	1
190	LAM	2	1	4	2	3	4	3	3	1	3	2	4	2	2	6	1	6	3	2	3	3	7	2	2	1	1	1	1	1	1	1	1	1	1	1	1	1	1	1	1	1	1	1	1	0	0	0	0	1	1	1	1	0	1	1	1	0	0	0	0	1	1	1	1	1	1	1
191	Cameroon	2	2	4	2	3	3	3	6	2	2	3	4	2	2	6	1	6	3	2	3	3	7	2	2	1	1	1	1	1	1	1	1	1	1	1	1	1	1	1	1	1	1	1	1	1	1	0	0	0	1	1	1	1	1	1	1	0	0	0	0	1	1	1	1	1	1	1
192	LAM	2	1	4	2	3	4	3	3	2	3	2	4	2	2	6	1	6	3	2	3	3	7	2	2	1	1	1	1	1	1	1	1	1	1	1	1	1	1	1	1	1	1	1	1	0	0	0	0	1	1	1	1	0	1	1	1	0	0	0	0	1	1	1	1	1	1	1
193	LAM	2	2	4	2	2	5	3	2	2	3	2	4	2	2	6	1	5	3	3	3	3	5	2	2	1	1	1	1	1	1	1	1	0	0	0	1	1	1	1	11	1	1	1	1	0	0	0	0	1	1	1	1	1	1	1	1	0	0	0	0	1	1	1	1	1	1	1
194	LAM	2	2	4	2	2	4	3	2	1	3	2	4	2	2	6	1	1	5	3	3	3	5	2	2	1	1	0	1	1	1	1	1	1	1	1	1	1	1	1	1	1	1	1	1	0	0	0	0	1	1	1	1	1	1	1	1	0	0	0	0	1	1	1	1	1	1	1
195	Cameroon	2	2	4	2	2	3	3	2	1		4	4	2	1	5	1	5	3	3	3	3	5	2	2	1	1	1	1	1	1	1	1	1	1	1	1	1	1	1	1	1	1	1	1	1	1	0	0	0	1	1	1	1	1	1	1	0	0	0	0	1	1	1	1	1	1	1
196	CAS-Delhi	2	4	2	2	3	2	3	5	1	1	4	4	2	1	5	1	7	3	3	6	4	8	2	2	1	1	1	0	0	0	0	0	1	1	1	1	1	1	1	1	1	1	1	1	1	1	0	0	1	1	1	1	1	1	1	1	1	0	0	0	0	1	1	1	1	1	1
197	CAS-Delhi	2	3	2	2	3	1	3	5	1	2	4	4	2	2	6	1	6	3	3	6	3	8	2	2	1	1	1	0	0	0	0	0	1	1	1	1	1	1	1	1	1	1	1	1	1	1	0	0	1	1	1	1	1	1	1	1	1	0	0	0	0	1	1	1	1	1	1
198	NEW1	2	2	4	2	3	2	3	5	2	2	4	4	2	2	5	1	7	3	2	3	3	6	2	2	1	0	0	1	1	1	1	1	1	1	1	1	1	1	1	1	1	1	1	1	1	1	1	1	1	1	1	1	0	0	0	1	0	0	0	0	1	1	1	1	1	1	1
199	CAS-Delhi	2	4	2	2	2	1	3	4	2	2	4	4	2	2	5	1	2	3	3	6	2	5	2	2	1	1	1	0	0	0	0	1	1	1	1	1	1	1	1	1	1	1	1	1	1	1	0	0	0	0	0	0	0	0	0	0	0	0	0	0	1	1	1	1	1	1	1
200	CAS-Delhi	2	3	2	2	4		3	4	2	2	4	4	2	2	5	1	5	3	3	6	3	8	2	2	1	1	1	0	0	0	0	0	1	1	1	1	1	1	1	1	1	1	1	1	1	1	0	0	1	1	1	1	1	1	1	1	1	0	0	0	0	1	1	1	1	1	1
201	CAS-Delhi	2	3	2	2	4		3	4	2	2	4	4	2	2	5	1	5	3	3	6	3		2	2	1	1	1	0	0	0	0	1	1	1	1	1	1	1	1	1	1	1	1	1	1	1	0	0	0	0	0	0	0	0	0	0	0	0	0	0	1	1	1	1	1	1	1
202	Beijing	2	6	2	2	2	3	3	5	3	1	4	4	4	2	6	1	7	3	3	6	4	6	2	2	0	0	0	0	0	0	0	0	0	0	0	0	0	0	0	0	0	0	0	0	0	0	0	0	0	0	0	0	0	0	0	0	0	0	0	0	1	1	1	1	1	1	1
203	NEW1	2	2	4	2	4	1	3	6	3	2	4	4	2	2	6	1	7	3	3	3	3	7	2	2	1	0	1	1	1	0	1	1	1	1	1	1	1	1	1	1	1	1	1	1	1	1	1	1	1	1	1	1	0	0	0	1	0	0	0	0	1	1	1	1	1	1	1
204	Uganda	2	3	3	2	4	3	3	3	3	2	4	4	2	1	3	1	5	3	3	3	3	6	2	2	1	1	1	1	1	1	1	1	1	1	1	1	1	1	1	1	1	1	1	1	1	1	1	1	1	1	1	1	1	1	1	1	0	0	0	0	1	1	1	1	1	1	1
205	NEW1	2	3	4	2	4	3	3	5	3	2	3	4	2	1	5	1	5	3	2	3	2	7	2	2	1	0	1	1	1	0	1	1	1	1	1	1	1	1	1	1	1	1	1	1	1	1	1	1	1	1	1	1	0	0	0	1	0	0	0	0	1	1	1	1	1	1	1
206	Beijing	2	4	4	2	5	3	2	5	3	6		4	2	1	6	1	5	3	3	6	3	8	2	2	0	0	0	0	0	0	0	0	0	0	0	0	0	0	0	0	0	0	0	0	0	0	0	0	0	0	0	0	0	0	0	0	0	0	0	0	1	1	1	1	1	1	1
207	Beijing	2	4	4	2	5	3	2	5	3	6		4	2	1	6	1	5	3	3	6	3	8	2	2	0	0	0	0	0	0	0	0	0	0	0	0	0	0	0	0	0	0	0	0	0	0	0	0	0	0	0	0	0	0	0	0	0	0	0	0	1	1	1	1	1	1	1
208	Beijing	2	4	4	2	5	3	2	4	3	6		4	2	2	6	1	5	3	3	6	3	8	2	2	0	0	0	0	0	0	0	0	0	0	0	0	0	0	0	0	0	0	0	0	0	0	0	0	0	0	0	0	0	0	0	0	0	0	0	0	1	1	1	1	1	1	1
209	Beijing	2	4	4	2	5	3	3	5	3	6		4	2	2	6	1	5	3	3	6	3	8	2	2	0	0	0	0	0	0	0	0	0	0	0	0	0	0	0	0	0	0	0	0	0	0	0	0	0	0	0	0	0	0	0	0	0	0	0	0	1	1	1	1	1	1	1
210	Beijing	2	4	4	2	5	3	4	5	3	6		4	2	2	6	1	5	3	3	6	3	8	2	2	0	0	0	0	0	0	0	0	0	0	0	0	0	0	0	0	0	0	0	0	0	0	0	0	0	0	0	0	0	0	0	0	0	0	0	0	1	1	1	1	1	1	1
211	Beijing	2	4	4	2	5	3	3	4	2	6	3	4	2	2	6	1	5	3	3	6	3	8	2	2	0	0	0	0	0	0	0	0	0	0	0	0	0	0	0	0	0	0	0	0	0	0	0	0	0	0	0	0	0	0	0	0	0	0	0	0	1	1	1	1	1	1	1
212	Beijing	2	3	4	2	3	4	3	4	2	6	5	4	3	3	6	1	6	3	3	4	4	5	2	2	0	0	0	0	0	0	0	0	0	0	0	0	0	0	0	0	0	0	0	0	0	0	0	0	0	0	0	0	0	0	0	0	0	0	0	0	1	1	1	1	1	1	1
213	Uganda	2	2	3	2	1	3	5	5	2	2	5	4	2	3	6	1	5	3	3	4	4	5	2	2	1	1	1	1	1	1	1	1	1	1	1	1	1	1	1	1	1	1	1	1	1	1	1	1	1	1	1	1	1	1	1	1	0	0	0	0	1	1	1	1	1	1	1
214	Uganda	2	2	3	2	1	3	5	5	2	2	5	4	2	3	6	1	5	3	3	4	4	5	2	2	1	1	1	1	1	1	1	1	1	1	1	1	1	1	1	1	1	1	1	1	1	1	1	1	1	1	1	1	1	1	1	1	0	0	0	0	1	1	1	1	1	1	1
215	Beijing	2	2	4	2	3	4	4	4	2	6	5	4	3	3	6	1	6	3	3	4	4	5	2	2	0	0	0	0	0	0	0	0	0	0	0	0	0	0	0	0	0	0	0	0	0	0	0	0	0	0	0	0	0	0	0	0	0	0	0	0	1	1	1	1	1	1	1
216	CAS-Delhi	2	2	4	2	3	6	4	4	2	1	5	4	2	3	6	1	4	3	3	4	4	5	2	4	1	1	1	0	0	0	0	1	1	1	1	1	1	1	1	1	1	1	1	1	1	1	0	0	0	0	0	0	0	0	0	0	0	0	0	0	1	1	0	0	1	1	1
217	CAS-Delhi	2	4	4	2	3	6	4	4	2	2	5	4	2	3	6	1	6	3	3	4	5	5	2	2	1	1	1	0	0	0	0	1	1	1	1	1	1	1	1	1	1	1	1	1	1	1	0	0	0	0	0	0	0	0	0	0	0	0	0	0	1	1	0	0	1	1	1
218	CAS-Delhi	2	2	3	2	1	6	4	4	2	2	5	4	2	3	6	1	5	3	3	4	5	5	2	4	1	0	1	1	1	1	1	1	1	1	1	1	1	1	1	1	1	1	1	1	1	1	1	1	1	1	1	1	0	0	0	1	0	0	0	0	1	1	1	1	1	1	1
219	Uganda	2	2	3	2	0	3	3	3	2	2	5	4	2	3	6	1	5	3	3	4	5	5	2	4	1	1	1	1	1	1	1	1	1	1	1	1	1	1	1	1	1	1	1	1	1	1	1	1	1	1	1	1	1	1	1	1	0	0	0	0	1	0	1	1	1	1	1
220	Beijing	2	3	4	2	3	3	2	4	2	5	4	4	4	3	6	1	6	3	3	3	5	7	2	3	0	0	0	0	0	0	0	0	0	0	0	0	0	0	0	0	0	0	0	0	0	0	0	0	0	0	0	0	0	0	0	0	0	0	0	0	1	1	1	1	1	1	1
221	Beijing	2	3	4	2	3	3	2	4	2	5	5	4	3	3	6	1	6	3	3	3	5	7	2	2	0	0	0	0	0	0	0	0	0	0	0	0	0	0	0	0	0	0	0	0	0	0	0	0	0	0	0	0	0	0	0	0	0	0	0	0	1	1	1	1	1	1	1
222	Beijing	2	3	4	2	3	3	2	4	2	5	5	4	4	3	6	1	6	3	3	4	5	7	2	2	0	0	0	0	0	0	0	0	0	0	0	0	0	0	0	0	0	0	0	0	0	0	0	0	0	0	0	0	0	0	0	0	0	0	0	0	1	1	1	1	1	1	1
223	LAM	2	3	4	2	3	3	2	5	2	2	2	4	2	2	6	1	4	3	2	4	5	7	2	2	1	1	0	0	0	1	1	1	1	1	1	1	1	1	1	1	1	1	1	1	0	0	0	0	1	1	1	1	1	1	1	1	0	0	0	0	1	1	1	1	1	1	1
224	Beijing	2	3	4	2	3	3	2	5	2	5	4	4	4	3	6	1	6	3	3	4	4	7	2	2	0	0	0	0	0	0	0	0	0	0	0	0	0	0	0	0	0	0	0	0	0	0	0	0	0	0	0	0	0	0	0	0	0	0	0	0	1	1	1	1	1	1	1
225	Haarlem	2	2	4	2	3	3	2	4	2	5	4	4	4	3	6	1	4	3	3	3	4	7	2	2	1	1	1	1	1	1	1	1	1	1	1	1	1	1	1	1	1	1	1	1	1	1	1	1	1	1	1	1	1	1	0	1	0	0	0	0	1	1	1	1	1	1	1
226	Beijing	2	3	4	2	2	3	4	4	2	5	4	4	4	3	6	1	4	3	3	4	4	5	2	2	0	0	0	0	0	0	0	0	0	0	0	0	0	0	0	0	0	0	0	0	0	0	0	0	0	0	0	0	0	0	0	0	0	0	0	0	1	1	1	1	1	1	1
227	Beijing	2	5	4	2	3	3	3	5	2	6	4	4	4	2	5	1	4	3	3	6	3	9		4	0	0	0	0	0	0	0	0	0	0	0	0	0	0	0	0	0	0	0	0	0	0	0	0	0	0	0	0	0	0	0	0	0	0	0	0	1	1	1	1	1	1	1
228	CAS-Delhi	2	5	2	2	4	7	5	4	2	2	4	4	2		5	1	5	3	3	5	3	9		4	1	1	1	0	0	0	0	0	1	1	1	1	1	1	1	1	1	1	1	1	1	1	0	0	1	1	1	1	1	1	1	1	1	0	0	0	0	1	1	1	1	1	1
229	Beijing	2	5	4	2	3	3	3	5	2	6	4	4	4	2	5	1	4	3	3	6	3	9		4	0	0	0	0	0	0	0	0	0	0	0	0	0	0	0	0	0	0	0	0	0	0	0	0	0	0	0	0	0	0	0	0	0	0	0	0	1	1	1	1	1	1	1
230	Beijing	2	5	4	2	3	3	4	5	2		4	4	4	2	5	1	4	3	3	6	3	9		4	0	0	0	0	0	0	0	0	0	0	0	0	0	0	0	0	0	0	0	0	0	0	0	0	0	0	0	0	0	0	0	0	0	0	0	0	1	1	1	1	1	1	1
231	CAS-Delhi	2	4	2	2	1	6	4	4	2	2	4	4	2	2	5	1	8	3	3	3	3	4		4	1	1	1	0	0	0	0	0	1	1	1	1	1	1	1	1	1	1	1	1	1	1	0	0	1	1	1	1	1	1	1	1	1	0	0	0	0	1	1	1	1	1	1
232	NEW1	2	2	4	3	4	2	3	5	2	2	2	4	2	1	5	1	5	3	2	3	4	1	2	2	1	0	1	1	1	0	1	1	1	1	1	1	1	1	1	1	1	1	1	1	1	1	1	1	1	1	1	1	0	0	0	1	0	0	0	0	1	1	1	1	1	1	1
233	CAS-Delhi	2	4	2	3	3	5	4	5	2	2	4	4	4	2	5	1	7	3	3	3	3	4	2	3	1	1	1	0	0	0	0	1	1	1	1	1	1	1	1	1	1	1	1	1	1	1	0	0	0	0	0	0	0	0	0	0	0	0	0	0	1	1	1	1	1	1	1
234	NEW1	2	2	4	3	3	2	3	5	2	2	3	4	2	1	5	1	6	3	3	3	3	6	2	2	1	0	1	1	1	0	1	1	1	1	1	1	1	1	1	1	1	1	1	1	1	1	1	1	1	1	1	1	0	0	0	1	0	0	0	0	1	1	1	1	1	1	1
235	NEW1	2	2	4	3	3	2	3	5	2	2	3	4	2	1	5	1	6	3	2	3	3	7	2	2	1	0	0	1	1	1	1	1	1	1	1	1	1	1	1	1	1	1	1	1	1	1	1	1	1	1	1	1	0	0	0	1	0	0	0	0	1	1	1	1	1	1	1
236	NEW1	2	2	4	3	3	2	3	3	2	3	3	4	2	1	5	1	5	3	3	3	3	5	2	1	1	0	0	1	1	1	1	1	1	1	1	1	1	1	1	1	1	1	1	1	1	1	1	1	1	1	1	1	0	0	0	1	0	0	0	0	1	1	1	1	1	1	1
237	CAS-Delhi	2	2	2	3	5	4	4	4	2	2	3	4	2	2	5	1	5	1	3	3	3	8	3	4	1	1	1	0	0	0	0	1	1	1	1	1	1	1	1	1	1	1	1	1	1	1	0	0	0	0	0	0	0	0	0	0	0	0	0	0	1	1	1	1	1	1	1
238	CAS-Delhi	2	4	2	2	3	7	4	2	2	2	2	4	6	2	5	1	9	3	3	3	3	5		4	1	1	1	0	0	0	0	1	1	1	1	1	1	1	1	1	1	1	1	1	1	1	0	0	0	0	0	0	0	0	0	0	0	0	0	0	1	1	1	1	1	1	1
239	CAS-Delhi	2	4	2	2	1	6	4	4	2	2	4	4	2	2	5	1	8	3	3	3	3	4		4	1	1	1	0	0	0	0	1	1	1	1	1	1	1	1	1	1	1	1	1	1	1	0	0	0	0	0	0	0	0	0	0	0	0	0	0	1	1	1	1	1	1	1
240	Cameroon	2	2	4	2	3	3	2	2		5	1	4	2	2	6	1	5	3	3	3	3		4	2	1	1	1	1	1	1	1	1	1	1	1	1	1	1	1	1	1	1	1	0	1	1	0	0	0	1	1	1	1	1	1	1	0	0	0	0	1	1	1	1	1	1	1
241	CAS-Delhi	2	4	2	2	3	7	5	4	2	2	4	4	2	2	5	1	6	3	3	6	3	8	4	4	1	1	1	0	0	0	0	1	1	1	1	1	1	1	1	1	1	1	1	1	1	1	0	0	0	0	0	0	0	0	0	0	0	0	0	0	1	1	1	1	1	1	1
242	CAS-Delhi	2	2	2	2	4	5	4	4	2	2	4	4	2	2	5	1	5	2	3	3	3	8		4	1	1	1	0	0	0	0	1	1	1	1	1	1	1	1	1	1	1	1	1	1	1	0	0	0	0	0	0	0	0	0	0	0	0	0	0	1	1	0	0	1	1	1
243	CAS-Delhi	2	5	2	2	4	7	5	4	2	2	4	4	2	2	5	1	5	3	3	5	3	9	4	4	1	1	1	0	0	0	0	1	1	1	1	1	1	1	1	1	1	1	1	1	1	1	0	0	0	0	0	0	0	0	0	0	0	0	0	0	1	1	1	1	1	1	1
244	NEW1	2	2	4	2	3	2	4	5		2	3	4	2	1	5	1	5	3	2	3	3	7		2	1	0	0	1	1	1	1	1	1	1	1	1	1	1	1	1	1	1	1	1	1	1	1	1	1	1	1	1	0	0	0	1	0	0	0	0	1	1	1	1	1	1	1
245	NEW1	2	2	4	2	3	2	3	5	2	2	3	4	2	1	5	1	6	3	2	3	3	7	2	2	1	0	0	1	1	1	1	1	1	1	1	1	1	1	1	1	1	1	1	1	1	1	1	1	1	1	1	1	0	0	0	1	0	0	0	0	1	1	1	1	1	1	1
246	CAS-Delhi	2	6	2	2	3	5	4	4	2			4	2	2	5	1	7	3	3	3	3	8		3	1	1	1	0	0	0	0	1	1	1	1	1	1	1	1	1	1	1	1	1	1	1	0	0	0	0	0	0	0	0	0	0	0	0	0	0	1	1	1	1	1	1	1
247	NEW1	2	2	4	2	3	2	3	5	2	2	3	4	2	1	5	1	6	3	2	3	3	6		2	1	0	1	1	1	0	1	1	1	1	1	1	1	1	1	1	1	1	1	1	1	1	1	1	1	1	1	1	0	0	0	1	0	0	0	0	1	1	1	1	1	1	1
248	LAM	1	4	4	2	5	5	2	3	2	2	1	4	1	2	6	1	5	3	3	3	2	8	4	2	1	1	0	0	0	1	1	1	1	1	1	1	1	1	1	1	1	1	1	1	0	0	0	0	1	1	1	1	1	1	1	1	0	0	0	0	1	1	1	1	1	1	1
249	Beijing	2	5	4	2	3	3	3	5	2	6	4	4	4	2	5	1	4	3	3	6	3	9	4	4	0	0	0	0	0	0	0	0	0	0	0	0	0	0	0	0	0	0	0	0	0	0	0	0	0	0	0	0	0	0	0	0	0	0	0	0	1	1	1	1	1	1	1
250	CAS-Delhi	2	1	2	2	3	6	2	4	2	2	3	4	2	2	5	1	7	3	3	6	2	7	4	4	1	1	1	0	0	0	0	1	1	1	1	1	1	1	1	1	1	1	1	1	1	1	0	0	0	0	0	0	0	0	0	0	0	0	0	0	1	1	1	1	1	1	1
251	Beijing	2	5	3	2	3	3	4	5	2	6	4	4	4	2	5	1	5	3	3	6	3	10	4	4	0	0	0	0	0	0	0	0	0	0	0	0	0	0	0	0	0	0	0	0	0	0	0	0	0	0	0	0	0	0	0	0	0	0	0	0	1	1	1	1	1	1	1
252	Beijing	2	5	4	2	3	3	3	5	2	6	4	4	4	2	5	1	4	3	3	6	3	9	4	4	0	0	0	0	0	0	0	0	0	0	0	0	0	0	0	0	0	0	0	0	0	0	0	0	0	0	0	0	0	0	0	0	0	0	0	0	1	1	1	1	1	1	1
253	Beijing	2		4	2	3	3	2	4	2	6	4	1	4	2	5	1	5	3	3	6	3		4	2	0	0	0	0	0	0	0	0	0	0	0	0	0	0	0	0	0	0	0	0	0	0	0	0	0	0	0	0	0	0	0	0	0	0	0	0	1	1	1	1	1	1	1
254	Beijing	2	5	4	2	3	3	2	4	2	6	4	4	4	2	5	1	5	3	3	6	3		4	2	0	0	0	0	0	0	0	0	0	0	0	0	0	0	0	0	0	0	0	0	0	0	0	0	0	0	0	0	0	0	0	0	0	0	0	0	1	1	1	1	1	1	1
255	NEW1	2	2	4	3	3	2	3	5	2	2	3	4	2	1	5	1	5	3	2	3	2	7	2	2	1	0	0	1	1	1	1	1	1	1	1	1	1	1	1	1	1	1	1	1	1	1	1	1	1	1	1	1	0	0	0	1	0	0	0	0	1	1	1	1	1	1	1
256	NEW1	2	2	5	3	5	2	3	5	2	2	3	4	2	1	6	1	5	3	2	3	3	7	2	2	1	0	0	1	1	1	1	1	1	1	1	1	1	1	1	1	1	1	1	1	1	1	1	1	1	1	1	1	0	0	0	1	0	0	0	0	1	1	1	1	1	1	1
257	CAS-Delhi	2	5	2	3	3	9	4	4	2	1	4	4	2	2	5	1	7	3	3	3	3	8	3	3	1	1	1	0	0	0	0	1	1	1	1	1	1	1	1	1	1	1	1	1	1	1	0	0	0	0	0	0	0	0	0	0	0	0	0	0	1	1	1	1	1	1	1
258	NEW1	2	2	4	3	3	2	3	5	2	2	3	3	2	1	5	1	5	2	2	3	5	7	2	2	1	0	0	1	1	1	1	1	1	1	1	1	1	1	1	1	1	1	1	1	1	1	1	1	1	1	1	1	0	0	0	1	0	0	0	0	1	1	1	1	1	1	1
259	CAS-Delhi	2	4	2	3	3	6	4	4		2	4	4	2	2	5	1	5	2	3	3	3	8	4	3	1	1	1	0	0	0	0	1	1	1	1	1	1	1	1	1	1	1	1	1	1	1	0	0	0	0	0	0	0	0	0	0	0	0	0	0	1	1	1	1	1	1	1
260	CAS-Delhi	2	5	2	3	5	6	4	4		2	4	4	2	2	5	1	7	2	2	3	3	8	2	3	1	1	1	0	0	0	0	1	1	1	1	1	1	1	1	1	1	1	1	1	1	1	0	0	0	0	0	0	0	0	0	0	0	0	0	0	1	1	1	1	1	1	1
261	CAS-Delhi	2		2	2	3	7	4	2		2	2	4	2	2	5	1	9	3	3	3	3	5		4	1	1	1	0	0	0	0	1	1	1	1	1	1	1	1	1	1	1	1	1	1	1	0	0	0	0	0	0	0	0	0	0	0	0	0	0	1	1	1	1	1	1	1
262	CAS-Delhi	2	4	2	2	3	7	4	2	2	2	2	4	6	2	5	1	9	3	3	3	3	5		4	1	1	1	0	0	0	0	1	1	1	1	1	1	1	1	1	1	1	1	1	1	1	0	0	0	0	0	0	0	0	0	0	0	0	0	0	1	1	0	0	1	1	1
263	Uganda	2	4	6	2	2	3	2	3	2	3	4	4	2	2	3	1	5	3	2	3	4	7		2	1	1	1	1	1	1	1	1	1	1	1	1	1	1	1	1	1	1	1	1	1	1	1	1	1	1	1	1	1	1	1	1	0	0	0	0	1	0	1	1	1	1	1
264	CAS-Delhi	2	4	2	2	3	8	4	4		2	2	4	6	2	5	1	9	3	3	3	3	5		4	1	1	1	0	0	0	0	1	1	1	1	1	1	1	1	1	1	1	1	1	1	1	0	0	0	0	0	0	0	0	0	0	0	0	0	0	1	1	0	0	1	1	1
265	Beijing	2	2	4	2	3	3	3	5	2	5	4	4	5	2	5	1	5	3	3	3	3	8		3	0	0	0	0	0	0	0	0	0	0	0	0	0	0	0	0	0	0	0	0	0	0	0	0	0	0	0	0	0	0	0	0	0	0	0	0	1	1	1	1	1	1	1
266	Haarlem	2	2	3	2	3		3	3	2	3	2	4	4	2	5	1	5	3	3	3	3	7	3	2	1	1	1	1	1	1	1	1	1	1	1	1	1	1	1	1	1	1	1	1	1	1	1	1	1	1	1	1	1	1	0	1	0	0	0	0	1	1	1	1	1	1	1
267	CAS-Delhi	2	5	2	2	3	5	4	4	2	2	3	4	2	2	5	1	7	3	3	3	3	8	3	3	1	1	1	0	0	0	0	1	1	1	1	1	1	1	1	1	1	1	1	1	1	1	0	0	0	0	0	0	0	0	0	0	0	0	0	0	1	1	1	1	1	1	1
268	CAS-Delhi	2	4	2	2	3	6	4	6	1	1	3	4	2	2	5	1	5	3	3	3	3	6	3	3	1	1	1	0	0	0	0	1	1	1	1	1	1	1	1	1	1	1	1	1	1	1	0	0	0	0	0	0	0	0	0	0	0	0	0	0	1	1	1	1	1	1	1
269	NEW1	2	2	4	2	3	2	3	5	2	2	3	4	2	1	5	1	6	3	2	3	3	7	2	2	1	0	0	1	1	1	1	1	1	1	1	1	1	1	1	1	1	1	1	1	1	1	1	1	1	1	1	1	0	0	0	1	0	0	0	0	1	1	1	1	1	1	1
270	CAS-Delhi	2	5	2	2	2	6	3	4	2	2	4	4	2	2	5	1	6	3	3	3	3	3		3	1	1	1	0	0	0	0	1	1	1	1	1	1	1	1	1	1	1	1	1	1	1	0	0	0	0	0	0	0	0	0	0	0	0	0	0	1	1	1	1	1	1	1
271	Ural1	2		4	2	2	9	2	3		2	4	4	4	2	5	1	1	3	3	3	3	8	3	2	1	1	1	1	1	1	1	1	1	1	1	1	1	1	1	1	1	1	1	1	1	1	1	1	1	1	1	1	0	0	0	1	0	0	0	0	1	1	1	1	1	1	1
272	NEW1	2		4	2	3	2	3	3	2	3	3	4	2	1	5	1	5	3	3	3	3	7	2	2	1	0	0	1	1	1	1	1	1	1	1	1	1	1	1	1	1	1	1	1	1	1	1	1	1	1	1	1	0	0	0	1	0	0	0	0	1	1	1	1	1	1	1
273	Cameroon	2	2	4	2	3	3	1	2	1	4	3	4	2	2	5	1	5	3	3	3	4	5	2	2	1	1	1	1	1	1	1	1	1	1	1	1	1	1	1	1	1	1	1	1	1	1	0	0	0	1	1	1	1	1	1	1	0	0	0	0	1	1	1	1	1	1	1
274	NEW1	2	2	4	2	3	2	3	5	2	2	3	4	2	1	5	1	6	3	2	3	3	7		2	1	0	0	1	1	1	1	1	1	1	1	1	1	1	1	1	1	1	1	1	1	1	1	1	1	1	1	1	0	0	0	1	0	0	0	0	1	1	1	1	1	1	1
275	Ural1	4	4	4	2	2	9	2	3		2	4	4	4	2	5	1	1	3	3	1	3	4	3	2	1	1	1	1	1	1	1	1	1	1	1	1	1	1	1	1	1	1	1	1	1	1	1	1	1	1	1	1	0	0	0	1	0	0	0	0	1	1	1	1	1	1	1
276	Cameroon	2	2	4	2	2	3	1	2	2	2	2	4	2	2	5	1	5	3	3	3	2	5		2	1	1	1	1	1	1	1	1	1	1	1	1	1	1	1	1	1	1	1	1	1	1	0	0	0	1	1	1	1	1	1	1	0	0	0	0	1	1	1	1	1	1	1
277	Cameroon	2	2	4	2	2	3	1	2	2	2	2	4	2	2	5	1	5	3	3	3	2	5		2	1	1	1	1	1	1	1	1	1	1	1	1	1	1	1	1	1	1	1	1	1	1	0	0	0	1	1	1	1	1	1	1	0	0	0	0	1	1	1	1	1	1	1
278	LAM	2	5	2	2	3	5	2	4				4	2	2	6	1	7	3	3	3	2	8		2	1	1	1	1	1	1	1	1	1	1	1	1	1	1	1	1	1	1	1	1	0	0	0	0	1	1	1	1	0	1	1	1	0	0	0	0	1	1	1	1	1	1	1
279	CAS-Delhi	2	3	2	2	3	6	3	6	2	1	3	4	2	2	5	1	6	3	3	5	3	6		3	1	1	1	0	0	0	0	1	1	1	1	1	1	1	1	1	1	1	1	1	1	1	0	0	0	0	0	0	0	0	0	0	0	0	0	0	1	1	1	1	1	1	1
280	NEW1	2	1	4	2	3	2	3	3		3	3	4	2	1	5	1	5	3	3	3	3	7	2	2	1	0	0	1	1	1	1	1	1	1	1	1	1	1	1	1	1	1	1	1	1	1	1	1	1	1	1	1	0	0	0	1	0	0	0	0	1	1	1	1	1	1	1
281	CAS-Delhi	2	2	2	3'	3	5	4	4	2	2	4	4	2	2	6	1	7	3	3	5	2	6	4	4	1	1	1	0	0	0	0	1	1	1	1	1	1	1	1	1	1	1	1	1	1	1	0	0	0	0	0	0	0	0	0	0	0	0	0	0	1	1	0	0	1	1	1
282	NEW1	2	2	4	2	3	2	3	5		2	3	4	2	1	5	1	7	3	2	3	3	7		2	1	0	0	1	1	1	1	1	1	1	1	1	1	1	1	1	1	1	1	1	1	1	1	1	1	1	1	1	0	0	0	1	0	0	0	0	1	1	1	1	1	1	1
283	NEW1	2	2	4	2	3	2	2	5		2	3	4	2	1	5	1	5	3	2	3	3	7	2	2	1	0	0	1	1	1	1	1	1	1	1	1	1	1	1	1	1	1	1	1	1	1	1	1	1	1	1	1	0	0	0	1	0	0	0	0	1	1	1	1	1	1	1
284	NEW1	2	2	4	2	3	2	2	5	2	2	3	4	2	1	5	1	6	3	2	3	3	7	2	2	1	0	0	1	1	1	1	1	1	1	1	1	1	1	1	1	1	1	1	1	1	1	1	1	1	1	1	1	0	0	0	1	0	0	0	0	1	1	1	1	1	1	1
285	Uganda	2	1	3	1	2	3	2	3	2	2	4	3	2	2	3	1	5	3	2	3		3	2	2	1	1	1	1	1	1	1	1	1	1	1	1	1	1	1	1	1	1	1	1	1	1	1	1	1	1	1	1	1	1	1	1	0	0	0	0	1	0	1	1	1	1	1
286	CAS-Delhi	2	4	2	2	3	3	3	4	2	2	4	4	2	2	5	1	7	3	3	5	3	8		3	1	1	1	0	0	0	0	1	1	1	1	1	1	1	1	1	1	1	1	1	1	1	0	0	0	0	0	0	0	0	0	0	0	0	0	0	1	1	0	0	1	1	1
287	Beijing	2	4	4	2	3	3	3	5		6	4	4	4	2	5	1	7	3	3	5	3	7	2	3	0	0	0	0	0	0	0	0	0	0	0	0	0	0	0	0	0	0	0	0	0	0	0	0	0	0	0	0	0	0	0	0	0	0	0	0	1	1	1	1	1	1	1
288	NEW1	2	2	4	2	3	2	2	5	2	2	3	4	2	1	5	1	4	3	2	3	5	7	2	2	1	0	0	1	1	1	1	1	1	1	1	1	1	1	1	1	1	1	1	1	1	1	1	1	1	1	1	1	0	0	0	1	0	0	0	0	1	1	1	1	1	1	1
289	Beijing	2	4	4	2	3	3	3	5		7	4	4	4	2	5	1	8	3	3	6	3			3	0	0	0	0	0	0	0	0	0	0	0	0	0	0	0	0	0	0	0	0	0	0	0	0	0	0	0	0	0	0	0	0	0	0	0	0	1	1	1	1	1	1	1
290	Beijing	2	4	4	2	3	3	3	5	2	6	4	4	4	2	5	1	7	3	3	3	5	7	2	3	0	0	0	0	0	0	0	0	0	0	0	0	0	0	0	0	0	0	0	0	0	0	0	0	0	0	0	0	0	0	0	0	0	0	0	0	1	1	1	1	1	1	1
291	Beijing	2	4	4	2	3	3	3	4	2	6	4	4	4	2	5	1	7	3	3	5	3	7		3	0	0	0	0	0	0	0	0	0	0	0	0	0	0	0	0	0	0	0	0	0	0	0	0	0	0	0	0	0	0	0	0	0	0	0	0	1	1	1	1	1	1	1
292	Beijing	2	4	4	2	3	2	2	5	2	6	4	4	4	2	5	1	7	3	3	5	3	7	2	3	0	0	0	0	0	0	0	0	0	0	0	0	0	0	0	0	0	0	0	0	0	0	0	0	0	0	0	0	0	0	0	0	0	0	0	0	1	1	1	1	1	1	1
293	CAS-Delhi	2	4	2	2	4	5	4	4	2	2	4	4	4	2	6	1	7	3	3	4	4	7		1	1	1	1	0	0	0	0	1	1	1	1	1	1	1	1	1	1	1	1	1	1	1	0	0	0	0	0	0	0	0	0	0	0	0	0	0	1	1	1	1	1	1	1
294	Beijing	2	4	4	2	3	3	3	5		6	4	4	4	2	5	1	5	3	3	5	3	8		3	0	0	0	0	0	0	0	0	0	0	0	0	0	0	0	0	0	0	0	0	0	0	0	0	0	0	0	0	0	0	0	0	0	0	0	0	1	1	1	1	1	1	1
295	Beijing	2	4	4	2	3	3	3	4		6	4	4	4	2	5	1	8	5	3	5	3			3	0	0	0	0	0	0	0	0	0	0	0	0	0	0	0	0	0	0	0	0	0	0	0	0	0	0	0	0	0	0	0	0	0	0	0	0	1	1	1	1	1	1	1
296	Beijing	2	4	4	2	3	3	3	4		6	4	4	4	2	5	1	5	3	3	5	3	8		3	0	0	0	0	0	0	0	0	0	0	0	0	0	0	0	0	0	0	0	0	0	0	0	0	0	0	0	0	0	0	0	0	0	0	0	0	1	1	1	1	1	1	1
297	Beijing	2	4	4	2	3	3	3	4	2	6	4	4	4	2	5	1	7	3	3	5	3	7		3	0	0	0	0	0	0	0	0	0	0	0	0	0	0	0	0	0	0	0	0	0	0	0	0	0	0	0	0	0	0	0	0	0	0	0	0	1	1	1	1	1	1	1
298	Beijing	2	4	4	2	3	3	3	4	2	6	4	4	4	2	5	1	5	3	3	5	3	8		3	0	0	0	0	0	0	0	0	0	0	0	0	0	0	0	0	0	0	0	0	0	0	0	0	0	0	0	0	0	0	0	0	0	0	0	0	1	1	1	1	1	1	1
299	Beijing	2	4	4	2	3	3	3	5	2	6	4	4	4	2	5	1	5	3	3	5	3	8	2	3	0	0	0	0	0	0	0	0	0	0	0	0	0	0	0	0	0	0	0	0	0	0	0	0	0	0	0	0	0	0	0	0	0	0	0	0	1	1	1	1	1	1	1
300	Beijing	2	4	4	3	3	3	3	5	2	6	4	4	4	2	5	1	5	3	3	5	3	8	2	3	0	0	0	0	0	0	0	0	0	0	0	0	0	0	0	0	0	0	0	0	0	0	0	0	0	0	0	0	0	0	0	0	0	0	0	0	1	1	1	1	1	1	1
301	Beijing	2	4	4	2	3	3	3	4		6	4	4	4	2	5	1	5	3	3	5	3	8		3	0	0	0	0	0	0	0	0	0	0	0	0	0	0	0	0	0	0	0	0	0	0	0	0	0	0	0	0	0	0	0	0	0	0	0	0	1	1	1	1	1	1	1
302	Beijing	2	4	4	2	3	2	2	5	2	6	4	4	4	2	5	1	7	3	3	5	3	7		3	0	0	0	0	0	0	0	0	0	0	0	0	0	0	0	0	0	0	0	0	0	0	0	0	0	0	0	0	0	0	0	0	0	0	0	0	1	1	1	1	1	1	1
303	CAS-Delhi	2	5	2	2	3	5	4	4	2	2	4	4	2	2	6	1	7	3	3	5	2	8	0	2	1	1	1	0	0	0	0	1	1	1	1	1	1	1	1	1	1	1	1	1	1	1	0	0	0	0	0	0	0	0	0	0	0	0	0	0	1	1	1	1	1	1	1
304	CAS-Delhi	2	4	2	2	3	5	4	5	2	2	4	4	2	2	5	1	8	3	3	4	3	8	2	3	1	1	1	0	0	0	0	1	1	1	1	1	1	1	1	1	1	1	1	1	1	1	0	0	0	0	0	0	0	0	0	0	0	0	0	0	1	1	0	0	1	1	1
305	NEW1	2	1	4	2	4	2	3	5	2	2	3	4	2	1	5	1	4	3	2	3	3	7	2	2	1	0	0	1	1	1	1	1	1	1	1	1	1	1	1	1	1	1	1	1	1	1	1	1	1	1	1	1	0	0	0	1	0	0	0	0	1	1	1	1	1	1	1
306	Haarlem	2	4	4	2	2	5	1	3	2	2	3	4	4	2	5	1	1	3	3	3	3	8		2	1	1	1	1	1	1	1	1	1	1	1	1	1	1	1	1	1	1	1	1	1	1	1	1	1	1	1	1	1	1	0	1	0	0	0	0	1	1	1	1	1	1	1
